# Fauna Europaea – Orthopteroid orders

**DOI:** 10.3897/BDJ.4.e8905

**Published:** 2016-06-29

**Authors:** Klaus-Gerhard Heller, Horst Bohn, Fabian Haas, Fer Willemse, Yde de Jong

**Affiliations:** ‡ Unaffiliated, Magdeburg, Germany; § Zoologische Staatssammlung Munchen, Munich, Germany; | Staatliches Museum fur Naturkunde, Stuttgart, Germany; ¶ Unaffiliated, Eygelshoven, Netherlands; # University of Amsterdam - Faculty of Science, Amsterdam, Netherlands; ¤ Museum für Naturkunde, Berlin, Germany

**Keywords:** Biodiversity Informatics, Fauna Europaea, Taxonomic indexing, Zoology, Biodiversity, Taxonomy, Orthoptera, *
Embiodea
*, *
Dermaptera
*, *
Phasmatodea
*, *
Dictyoptera
*, *
Mantodea
*, *
Blattaria
*, *
Isoptera
*

## Abstract

*Fauna Europaea* provides a public web-service with an index of scientific names (including important synonyms) of all extant European terrestrial and freshwater animals, their geographical distribution at the level of countries and major islands (west of the Urals and excluding the Caucasus region), and some additional information. The *Fauna Europaea* project comprises about 230,000 taxonomic names, including 130,000 accepted species and 14,000 accepted subspecies, which is much more than the originally projected number of 100,000 species. *Fauna Europaea* represents a huge effort by more than 400 contributing specialists throughout Europe and is a unique (standard) reference suitable for many users in science, government, industry, nature conservation and education.

The “Orthopteroid orders“ is one of the 58 *Fauna Europaea* major taxonomic groups. It contains series of mostly well-known insect orders: *Embiodea* (webspinners), *Dermaptera* (earwigs), *Phasmatodea* (walking sticks), Orthoptera s.s. (grasshoppers, crickets, bush-crickets) and *Dictyoptera* with the suborders *Mantodea* (mantids), *Blattaria* (cockroaches) and *Isoptera* (termites).

For the Orthopteroid orders, data from 35 families containing 1,371 species are included in this paper.

## Introduction

In 1998 the European Commission published the European Community Biodiversity Strategy, providing a framework for development of Community policies and instruments in order to comply with the Convention on Biological Diversity. This Strategy recognises the current incomplete state of knowledge at all levels of biodiversity, a state which makes a successful implementation of the Convention difficult. *Fauna Europaea* was conceived to contribute to this Strategy by supporting one of the main themes: to identify and catalogue the components of European biodiversity, with the cataloguing implemented as a taxonomic and faunistic database serving as a basic tool for scientific documentation and discovery, environmental management, and conservation policies/priorities.

With regard to biodiversity in Europe, science and policies depend on sufficient knowledge of the relevant components. The assessment of biodiversity, monitoring changes, sustainable exploitation of biodiversity, as well as much legislative work depend upon a validated taxonomic overview, in which *Fauna Europaea* plays a major role by providing a web-based information infrastructure with an index of scientific names (including the most important synonyms) of all living European multicellular terrestrial and freshwater animals, their geographical distribution at the level of countries and major islands, and some relevant additional information. In this sense, the *Fauna Europaea* database provides a unique reference for many user-groups such as scientists, governments, industries, conservation communities and educational programs.

*Fauna Europaea* (FaEu) kicked off in 2000 as an EC-FP5 four-year project, delivering its first release in 2004 (Jong de et al. 2014). *Fauna Europaea* has continuously been updated, and after a further decade of steady progress, to efficiently disseminate the results of *Fauna Europaea* and to properly credit the *Fauna Europaea* contributors, modern e-publishing tools are being applied to prepare data papers on all 58 major taxonomic groups. For this purpose a special Biodiversity Data Journal Series has been compiled, called Contributions on Fauna Europaea (see also: Pensoft News item 17 Dec 2014). This work was initiated during the ViBRANT project and is further supported by the recently started EU BON project.

In the EU BON project also further steps will be made to implement *Fauna Europaea* as a basic tool and standard reference for biodiversity research and as a means to facilitate taxonomic expertise evaluation and management in Europe, including its contributions to the European Taxonomic Backbone (PESI / EU-nomen) project (Jong de et al. 2015).

This paper is the first publication from the *Fauna Europaea* Orthopteroid Orders data sector as a BDJ data paper in the *Fauna Europaea* series. The paper is dedicated to Fer Willemse, prominent orthopterologist, respected member of our Fauna Europaea community and co-author of this paper, who passed away in 2009.

## General description

### Purpose

*Fauna Europaea* is a database of the scientific names and distributions (at national or in some cases regional level) of all currently known extant multicellular European terrestrial and freshwater animal species. The database has been assembled by a large network of taxonomic specialists. An extended description of the *Fauna Europaea* project can be found in Jong de et al. (2014). A summary is given in the sections below.

The Orthopteroid Orders is one of the 58 *Fauna Europaea* major taxonomic groups, covering 1,371 species. The data were acquired and checked by a network of 4 specialists (Tables 1, 2).

### Additional information

*Introduction Orthopteroid Orders*

Under the name “Orthopteroid orders“ in the wide sense as used here all orders (except Plecoptera: stoneflies) are combined which make up the group (superorder) Polyneoptera (e.g. Grimaldi and Engel 2004): *Embiodea* (webspinners), *Dermaptera* (earwigs), *Phasmatodea* (walking sticks), Orthoptera s.s. (grasshoppers, crickets, bush-crickets) and *Dictyoptera* with the suborders *Mantodea* (mantids), *Blattaria* (cockroaches) and *Isoptera* (termites), for which information can be easily obtained in the internet (e.g. wikipedia).

A compilation of references, used for the preparation of the first version, is appended under 'Additional Information' below.

## Project description

### Title

This BDJ data paper includes the taxonomic indexing efforts in the *Fauna Europaea* on European Orthoptera covering the first two versions of *Fauna Europaea* worked (up to version 2.6).

### Personnel

Taxonomic framework of *Fauna Europaea* includes partner institutes, which together with a number of local- and citizen scientists provide the taxonomic expertise and faunistic quality assurance and take care of data collation.

Every taxonomic group is covered by at least one Group Coordinator responsible for the supervision and integrated input of taxonomic and occurrence data of a particular group. For Orthoptera the responsible Group Coordinators is Klaus-Gerhard Heller.

The *Fauna Europaea* checklist would not have reached its current level of completion without the input from several groups of specialists. The formal responsibility of collating and delivering the data for relevant families has resided with the appointed Taxonomic Specialists (see Table 1). Associate Specialists deserve due credit for their important contributions at various levels, including particular geographic regions or (across) taxonomic groups (see Table 2).

Data management tasks were taken care about by the *Fauna Europaea* project bureau. During the project phase (until 2004) a network of principal partners took care about diverse management tasks: Zoological Museum Amsterdam (general management & system development), Zoological Museum of Copenhagen (data collation), National Museum of Natural History in Paris (data validation) and Museum and Institute of Zoology in Warsaw (Newly Associated States [NAS] extension). From the formal termination of the project in 2004 to 2013, all tasks were taken over by the Zoological Museum Amsterdam.

### Study area description

**Figure 1.**
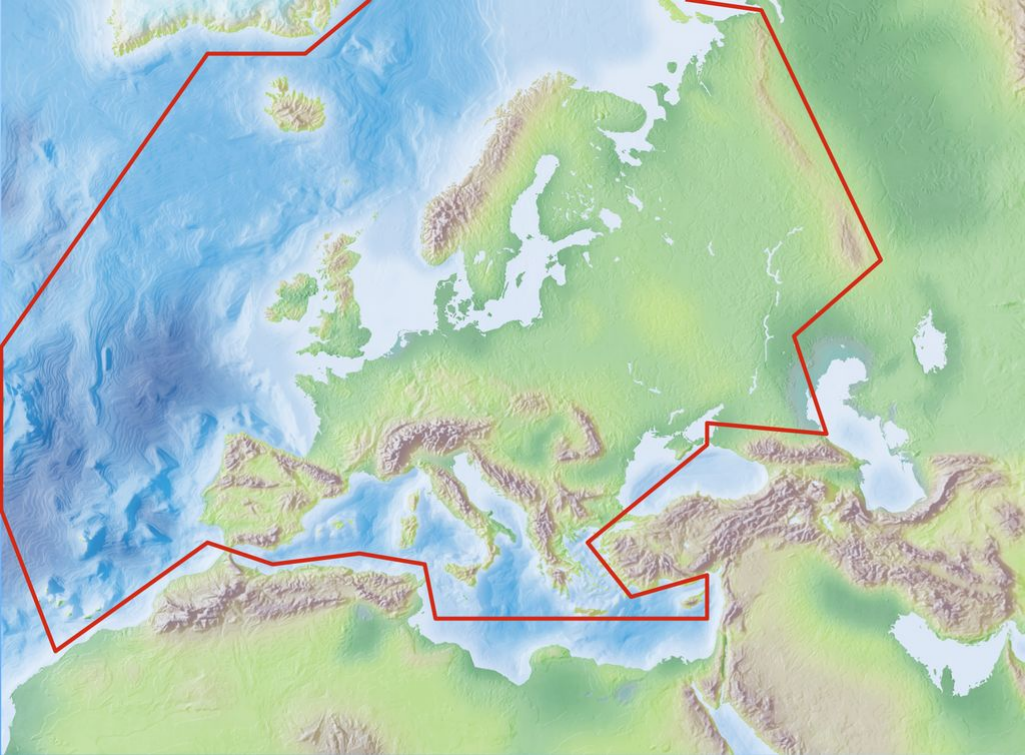
*Fauna Europaea* geographic coverage ('minimal Europe').

The study area covers the western Palaearctic, including the European mainland, Great Britain, the Macaronesian islands, Cyprus, Faroe Islands, Iceland, Svalbard, Franz Josef Land and Novaya Zemlya, but excluding (non-European) Turkey, the Caucasus, western Kazakhstan, the Arabian Peninsula and North Africa (Fig. 1).

### Design description

*Standards*. Group Coordinators and taxonomic specialists have been delivering the (sub)species names according to strict standards. The names provided by *Fauna Europaea* are *scientific names*. The taxonomic scope includes issues like, (1) the definition of criteria used to identify the accepted species-group taxa, (2) the hierarchy (classification scheme) for the accommodation of all accepted (sub)species, (3) relevant synonyms, and (4) the correct nomenclature. The *Fauna Europaea* 'Guidelines for Group Coordinators and Taxonomic Specialists' (Suppl. material 1) include the standards, protocols, scope and geographical limits and provide the instructions for the more than 400 taxonomic specialists contributing to the project, following the provisions of the International Code of Zoological Nomenclature.

**Figure 2.**
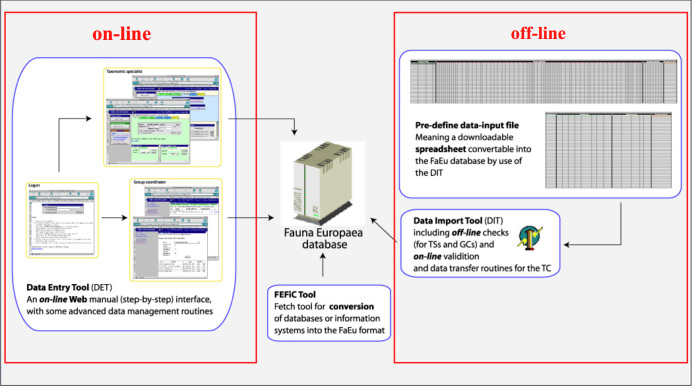
*Fauna Europaea* on-line (browser interfaces) and off-line (spreadsheets) data entry tools.

*Data management*. The data records could either be entered offline into a preformatted MS-Excel worksheet or directly into the *Fauna Europaea* transaction database using an online browser interface (Fig. 2). The data servers were hosted at the Academic Informatics Center of the University of Amsterdam (SARA/Vancis). Since 2013 the data servers are hosted at the Museum für Naturkunde in Berlin, and a new data entry (update) tool is under development.

*Data set*. The *Fauna Europaea* basic data set consists of: accepted (sub)species names (including authorship), synonym names (including authorship), a taxonomic hierarchy / classification, misapplied names (including misspellings and alternative taxonomic views), homonym annotations, expert details, European distribution (at country level or major islands), global distribution (only for European species), taxonomic reference (optional), occurrence reference (optional).

### Funding

*Fauna Europae*a was funded by the European Commission under the Fifth Framework Programme and contributed to the Support for Research Infrastructures work programme with Thematic Priority Biodiversity (EVR1-1999-20001) for a period of four years (1 March 2000 – 1 March 2004), including a short 'NAS extension', allowing EU candidate accession countries to participate. Follow-up support was given by the EC-FP5 EuroCAT project (EVR1-CT-2002-20011), by the EC-FP6 ENBI project (EVK2-CT-2002-20020), by the EC-FP6 EDIT project (GCE 018340), by the EC-FP7 PESI project (RI-223806) and by the EC-FP7 ViBRANT project (RI-261532). Continued management and hosting of the *Fauna Europaea* services was supported by the University of Amsterdam (Zoological Museum Amsterdam) and SARA/Vancis. Recently, the hosting of *Fauna Europaea* was taken over by the Museum für Naturkunde in Berlin, supported by the EC-FP7 EU BON project (grant agreement ENV-308454).

Additional support for preparing the Orthoptera data set was received through the numerous institutions allowing for the proper allocation of time by the contributing taxonomic specialists.

## Sampling methods

### Study extent

See spatial coverage and geographic coverage descriptions.

### Sampling description

*Fauna Europaea* data have been assembled by principal taxonomic experts, based on their individual expertise, which includes literature study, collection research, and field observations. In total 476 taxonomic specialists contributed taxonomic and/or faunistic information for *Fauna Europaea.* The vast majority of the experts are from Europe (including EU non-member states). As a unique feature, *Fauna Europaea* funds were set aside for paying/compensating for the work of taxonomic specialists and Group Coordinators (around five Euro per species).

To facilitate data transfer and data import, sophisticated on-line (web interfaces) and off-line (spreadsheets) data-entry routines were built, well integrated within an underlying central *Fauna Europaea* transaction database (see Fig. 2). This includes advanced batch data import routines and utilities to display and monitor the data processing within the system. In retrospect, it seems that the off-line submission of data was probably the best for bulk import during the project phase, while the on-line tool was preferred to enter modifications in later versions. This system worked well until 2013, but will be replaced by a new system in 2016.

A first release of the *Fauna Europaea* index via the web-portal has been presented at 27^th^ of September 2004, whereas the most recent release (version 2.6.2) was launched at 29 August 2013. An overview of *Fauna Europaea* releases can be found here: http://www.faunaeur.org/about_fauna_versions.php.

### Quality control

*Fauna Europaea* data are unique in the sense that they are fully expert based. Selecting leading experts for all groups provided a principal assurance of the systematic reliability and consistency of the *Fauna Europaea* data.

Furthermore, all *Fauna Europaea* data sets are intensively reviewed at regional and thematic validation meetings, at review sessions on taxonomic symposia (for some groups), by *Fauna Europaea* Focal Points (during the FaEu-NAS and PESI projects) and by various end-users sending annotations using the web form at the web-portal. Additional validation on gaps and correct spellings was effected by the validation office the National Museum of Natural History in Paris.

Checks on technical and logical correctness of the data were implemented by the data entry tools, including around 50 'Taxonomic Integrity Rules'. This validation tool proved to be of considerable value for both the taxonomic specialists and project management, and significantly contributed to the preparation of a remarkably clean and consistent data set.

This thorough review procedure makes *Fauna Europaea* the most scrutinised data set in its domain. In general we expected to get taxonomic data for 99.3% of the known European fauna directly after the initial release of *Fauna Europaea* (Jong de et al. 2014). The faunistic coverage is not quite as good, but is nevertheless 90-95% of the total fauna. For the Orthoptera, the taxonomic completeness is difficult to estimate (see also Heller et al. 1998). The total number of existing Orthoptera species in Europe is supposed to be around 20% higher compared to the current knowledge level (see Table 1).

To optimise the use and implementation of a uniform and correct nomenclature, a cross-referencing of the Fauna Europaea Orthopteroid data-set with relevant taxonomic resources is recommended, also supporting the global efforts on establishing a global taxonomic resolution service, provisionally called 'Global Names Architecture' (Pyle and Michel 2008, Jong de et al. 2015). Applicable nomenclature databases specifically dedicated to Orthopteroid species includes: Orthoptera Species File, Phasmida Species File Online, Dermaptera Species File, Cockroach Species File Online, Embioptera Species File Online, and Mantodea Species File Online. As a preparation, a semi-automatic validation on selected Orthopteroid species data files has been carried out, with help of the responsible curators, using the PESI Taxon Match Tool and LifeWatch Backbone services (Suppl. material 3). The results are cross-indexed with the Global Names Index and could be used to further integrate the Fauna Europaea and Orthopteroid species databases and web-services.

### Step description

By evaluating team structure and life cycle procedures (data-entry, validation, updating, etc.), clear definitions of roles of users and user-groups according to the taxonomic framework were established, including ownership and read/write privileges, and and their changes during the project's life-cycle. In addition, guidelines on common data exchange formats and codes have been issued (see also Suppl. material 1).

## Geographic coverage

### Description

Species and subspecies distributions in *Fauna Europaea* are registered at least at the level of (political) country. For this purpose the FaEu geographical system basically follows the TDWG standards (see: Suppl. material 1). The area studied covers the western Palaearctic west of the Urals, including the European mainland, Great Britain, the Macaronesian islands, Cyprus, Faroe Islands, Iceland, Svalbard, Franz Josef Land and Novaya Zemlya, but excluding (non-European) Turkey, the Caucasus, western Kazakhstan, the Arabian Peninsula and North Africa (see Fig. 1).

The focus is on species (or subspecies) of European multicellular animals of terrestrial and freshwater environments. Species in brackish waters, occupying the marine/freshwater or marine/terrestrial transition zones, are generally excluded.

### Coordinates

Mediterranean (N 35°) and Arctic Islands (N 82°) Latitude; Atlantic Ocean (Mid-Atlantic Ridge) (W 30°) and Ural (E 60°) Longitude.

## Taxonomic coverage

### Description

**Figure 3.**
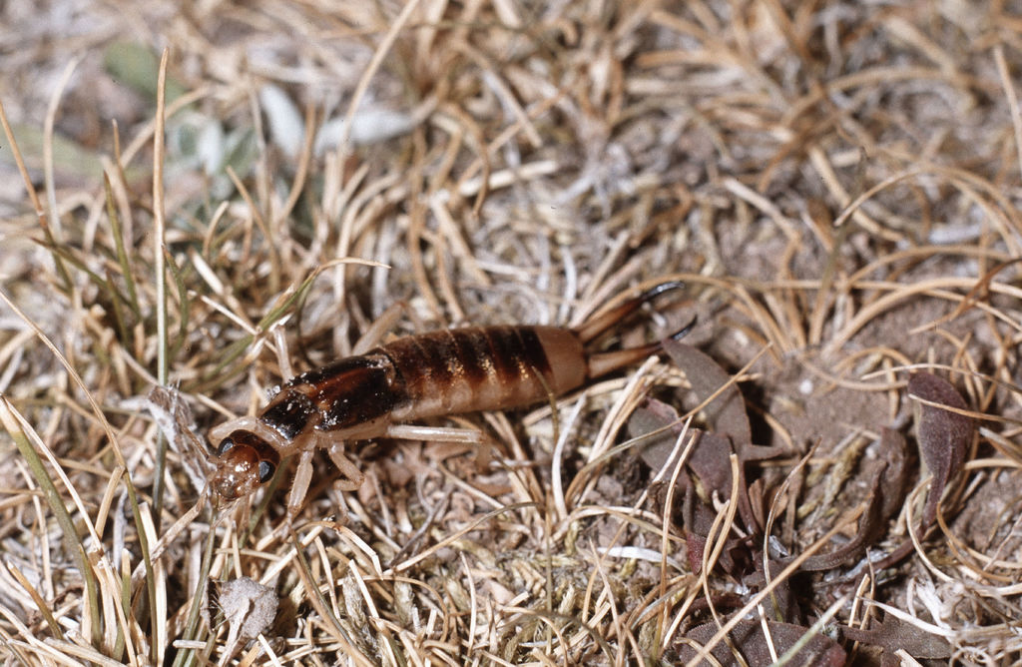
Order Dermaptera, Family Labiduridae, *Labidura riparia* (Pallas 1773). Location: Greece, river Tauropos between Arta and Karpenision. Photo by Klaus-Gerhard Heller.

**Figure 4.**
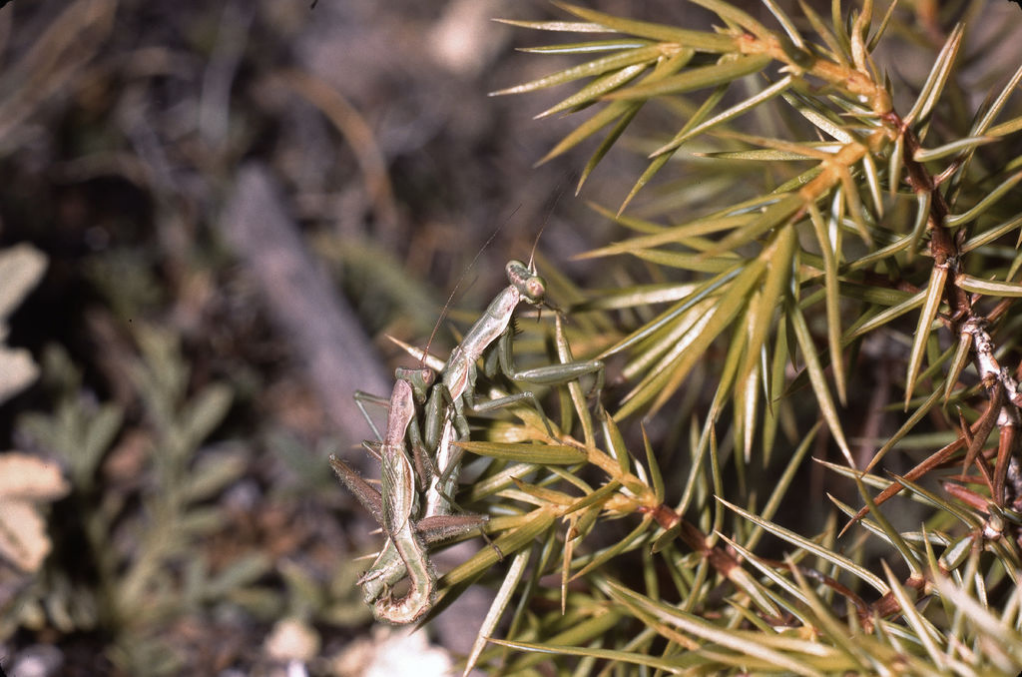
Order Dictyoptera, Suborder Mantodea, Family Mantidae, *Pseudoyersinia paui* (Bolivar 1898): Mating. Location: Spain, Bovalar near Morella. Photo by Klaus-Gerhard Heller.

**Figure 5.**
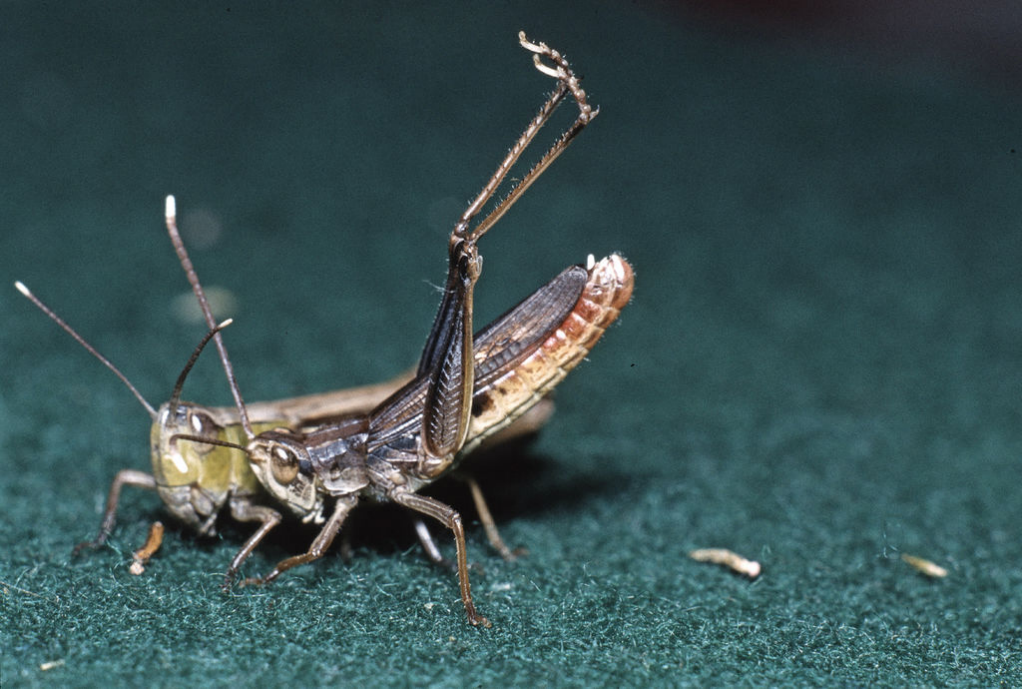
Order Orthoptera, Suborder Caelifera, Superfamily Acridoidea, Family Acrididae, *Chorthippus lacustris* La Greca & Messina 1975: Courtship. Location: Greece, Ioannina. Photo by Klaus-Gerhard Heller.

**Figure 6.**
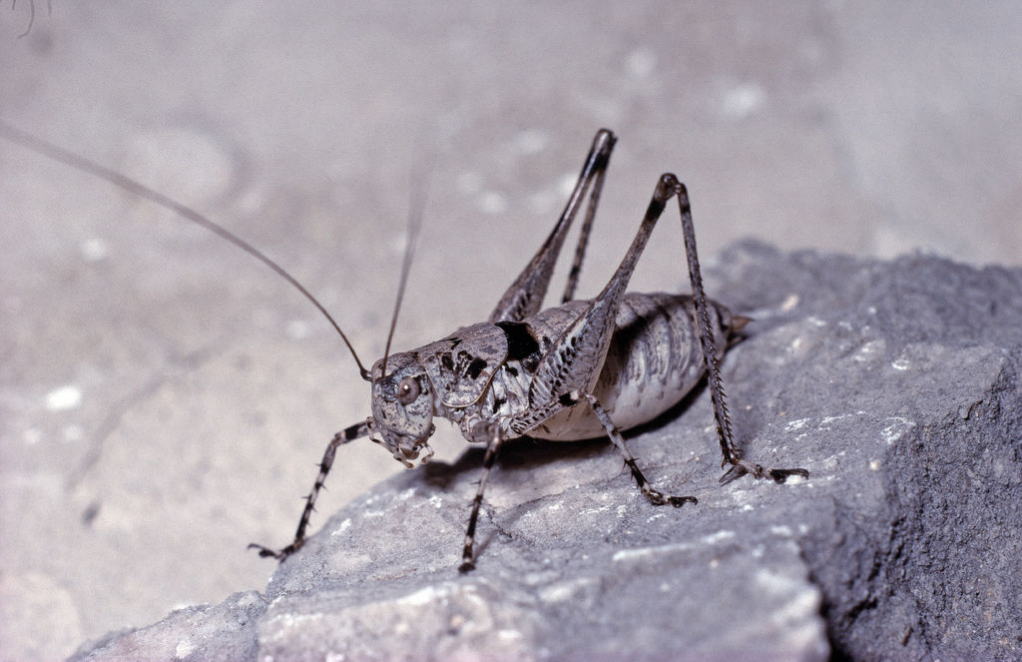
Order Orthoptera, Suborder Ensifera, Superfamily Tettigonioidea, Family Tettigoniidae, *Rhacocleis crypta* F. Willemse & L. Willemse 2005. Location: Greece, Mount Gavrogo near Arta. Photo by Klaus-Gerhard Heller.

**Figure 7.**
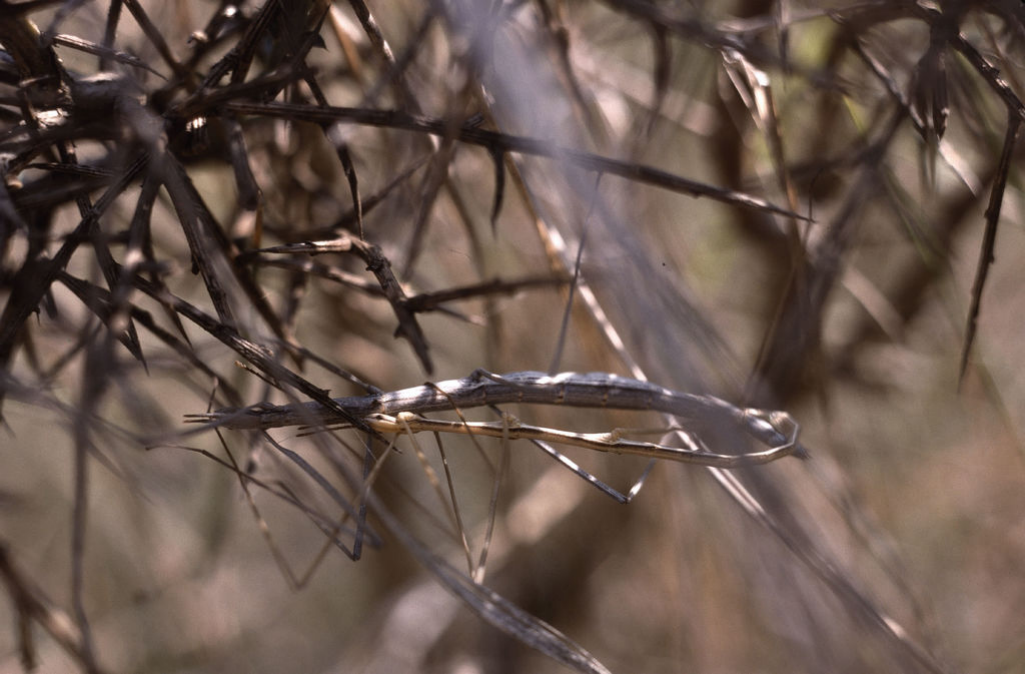
Order Phasmatodea, Family Heteronemiidae, *Pijnackeria lucianae* Scali, Milani & Passamonti 2013: Mating. Location: Spain, Ibi near Alcoy. Photo by Klaus-Gerhard Heller.

The *Fauna Europaea* database contains the scientific names of all living European land and freshwater animal species, including numerous infra-groups and synonyms. More details about the conceptual background of *Fauna Europaea* and standards followed are described above and in the project description paper(s). Figs 3, 4, 5, 6, 7

**Figure 8.**
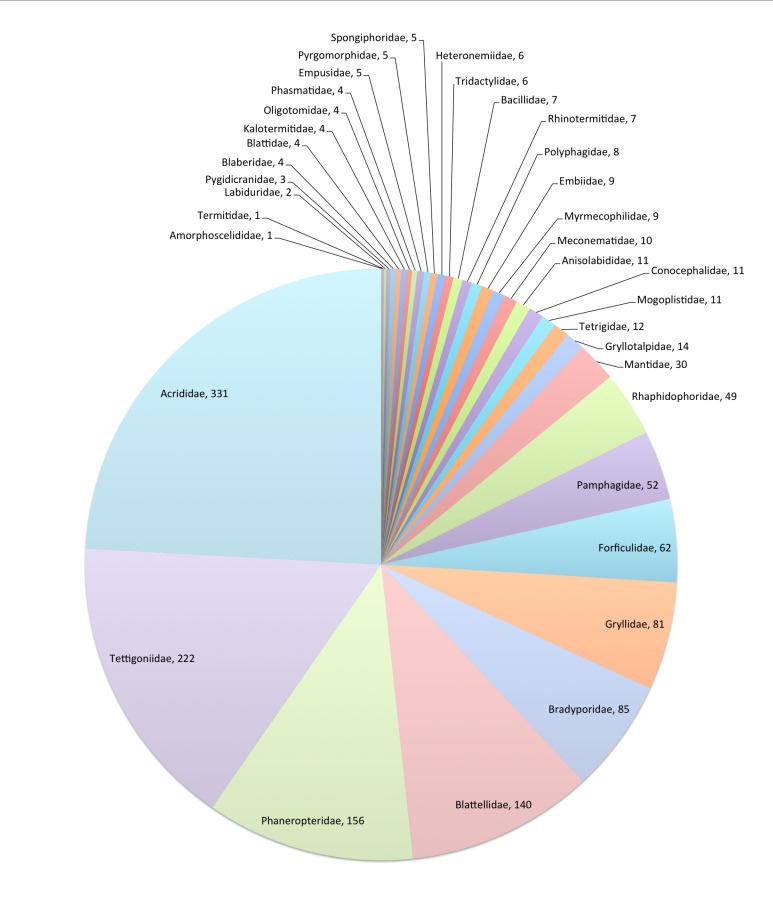
FaEu Orthoptera species per family. See Table 1 for family statistics. For full resolution see Suppl. material 2. Further details on the *Fauna Europaea* Orthoptera classificationcan be found here: http://www.faunaeur.org/experts.php?id=55.

This data paper covers the Orthopteriod Orders content of *Fauna Europaea*, including 35 families, 1,371 species, 48 subspecies and 201 (sub)species synonyms (see Fig. 8). Higher ranks are given below, the species list can be downloaded (see: Data resources).

### Taxa included

**Table:** 

Rank	Scientific Name	
kingdom	Animalia	
subkingdom	Eumetazoa	
phylum	Arthropoda	
subphylum	Hexapoda	
class	Insecta	
order	Dermaptera	
family	Anisolabididae	
subfamily	Carcinophorinae	
subfamily	Pseudisolabiinae	
family	Forficulidae	
subfamily	Allodahlinae	
subfamily	Anechurinae	
subfamily	Forficulinae	
family	Labiduridae	
subfamily	Labidurinae	
subfamily	Nalinae	
family	Pygidicranidae	
subfamily	Anataelinae	
family	Spongiphoridae	
subfamily	Isolaboidinae	
subfamily	Labiinae	
subfamily	Spongiphorinae	
order	Dictyoptera	
suborder	Blattodea	
family	Blaberidae	
subfamily	Blaberinae	
subfamily	Oxyhaloinae	
subfamily	Pycnoscelinae	
family	Blattellidae	
subfamily	Blattellinae	
subfamily	Ectobiinae	
subfamily	Pseudophyllodromiinae	
family	Blattidae	
subfamily	Blattinae	
family	Polyphagidae	
subfamily	Euthyrrhaphinae	
subfamily	Polyphaginae	
suborder	Isoptera	
family	Kalotermitidae	
family	Rhinotermitidae	
family	Termitidae	
suborder	Mantodea	
family	Amorphoscelididae	
family	Empusidae	
family	Mantidae	
order	Embioptera	
family	Embiidae	
family	Oligotomidae	
order	Orthoptera	
suborder	Caelifera	
superfamily	Acridoidea	
family	Acrididae	
subfamily	Acridinae	
subfamily	Calliptaminae	
subfamily	Catantopinae	
subfamily	Cyrtacanthacridinae	
subfamily	Dericorythinae	
subfamily	Egnatiinae	
subfamily	Eyprepocnemidinae	
subfamily	Gomphocerinae	
subfamily	Oedipodinae	
subfamily	Tropidopolinae	
family	Pamphagidae	
subfamily	Akicerinae	
subfamily	Pamphaginae	
family	Pyrgomorphidae	
subfamily	Pyrgomorphinae	
superfamily	Tetrigoidea	
family	Tetrigidae	
superfamily	Tridactyloidea	
family	Tridactylidae	
suborder	Ensifera	
superfamily	Grylloidea	
family	Gryllidae	
subfamily	Gryllinae	
subfamily	Gryllomorphinae	
subfamily	Nemobiinae	
subfamily	Oecanthinae	
subfamily	Trigonidiinae	
family	Gryllotalpidae	
family	Mogoplistidae	
family	Myrmecophilidae	
superfamily	Rhaphidophoroidea	
family	Rhaphidophoridae	
subfamily	Dolichopodainae	
subfamily	Rhaphidophorinae	
subfamily	Troglophilinae	
superfamily	Tettigonioidea	
family	Bradyporidae	
family	Conocephalidae	
family	Meconematidae	
family	Phaneropteridae	
family	Tettigoniidae	
order	Phasmatodea	
family	Bacillidae	
family	Heteronemiidae	
family	Phasmatidae	

## Temporal coverage

**Living time period:** Currently living.

### Notes

Currently living animals in stable populations, largely excluding (1) rare/irregular immigrants, intruder or invader species, (2) accidental or deliberate releases of exotic (pet) species, (3) domesticated animals, (4) foreign species imported and released for bio-control or (5) foreign species largely confined to hothouses.

## Usage rights

### Use license

Open Data Commons Attribution License

### IP rights notes

*Fauna Europaea* data are licensed under CC BY SA version 4.0. The experts keep property rights over their data, initially covered under the FaEu/SMEBD conditions. For more copyrights and citation details see: http://www.faunaeur.org/copyright.php.

For correct use and citing of the Orthopteroid data sets (Suppl. material 3), please check the relevant websites.

## Data resources

### Data package title

Fauna Europaea - Orthopteroids

### Resource link

http://www.faunaeur.org/Data_papers/FaEu_Orthopteroids_2.6.2.zip

### Alternative identifiers

http://www.faunaeur.org/full_results.php?id=11883

### Number of data sets

2

### Data set 1.

#### Data set name

Fauna Europaea - Orthopteroids version 2.6.2 - species

#### Data format

CSV

#### Number of columns

25

#### Character set

UTF-8

#### Download URL

http://www.faunaeur.org/Data_papers/FaEu_Orthopteroids_2.6.2.zip

#### 

**Data set 1.** 

Column label	Column description
datasetName	The name identifying the data set from which the record was derived (http://rs.tdwg.org/dwc/terms/datasetName).
version	Release version of data set.
versionIssued	Issue data of data set version.
rights	Information about rights held in and over the resource (http://purl.org/dc/terms/rights).
rightsHolder	A person or organization owning or managing rights over the resource (http://purl.org/dc/terms/rightsHolder).
accessRights	Information about who can access the resource or an indication of its security status (http://purl.org/dc/terms/accessRights).
taxonID	An identifier for the set of taxon information (http://rs.tdwg.org/dwc/terms/taxonID).
parentNameUsageID	An identifier for the name usage of the direct parent taxon (in a classification) of the most specific element of the scientificName (http://rs.tdwg.org/dwc/terms/parentNameUsageID).
scientificName	The full scientific name, with authorship and date information if known (http://rs.tdwg.org/dwc/terms/scientificName).
acceptedNameUsage	The full name, with authorship and date information if known, of the currently valid (zoological) taxon (http://rs.tdwg.org/dwc/terms/acceptedNameUsage).
originalNameUsage	The original combination (genus and species group names), as firstly established under the rules of the associated nomenclaturalCode (http://rs.tdwg.org/dwc/terms/originalNameUsage).
family	The full scientific name of the family in which the taxon is classified (http://rs.tdwg.org/dwc/terms/family).
familyNameId	An identifier for the family name.
genus	The full scientific name of the genus in which the taxon is classified (http://rs.tdwg.org/dwc/terms/genus).
subgenus	The full scientific name of the subgenus in which the taxon is classified. Values include the genus to avoid homonym confusion (http://rs.tdwg.org/dwc/terms/subgenus).
specificEpithet	The name of the first or species epithet of the scientificName (http://rs.tdwg.org/dwc/terms/specificEpithet).
infraspecificEpithet	The name of the lowest or terminal infraspecific epithet of the scientificName, excluding any rank designation (http://rs.tdwg.org/dwc/terms/infraspecificEpithet).
taxonRank	The taxonomic rank of the most specific name in the scientificName (http://rs.tdwg.org/dwc/terms/infraspecificEpithet).
scientificNameAuthorship	The authorship information for the scientificName formatted according to the conventions of the applicable nomenclaturalCode (http://rs.tdwg.org/dwc/terms/scientificNameAuthorship).
authorName	Author name information.
namePublishedInYear	The four-digit year in which the scientificName was published (http://rs.tdwg.org/dwc/terms/namePublishedInYear).
Brackets	Annotation if authorship should be put between parentheses.
nomenclaturalCode	The nomenclatural code under which the scientificName is constructed (http://rs.tdwg.org/dwc/terms/nomenclaturalCode).
taxonomicStatus	The status of the use of the scientificName as a label for a taxon (http://rs.tdwg.org/dwc/terms/taxonomicStatus).
resourceDescription	An account of the resource, including a data-paper DOI (http://purl.org/dc/terms/description).

### Data set 2.

#### Data set name

Fauna Europaea - Orthopteroids version 2.6.2 - hierarchy

#### Data format

CSV

#### Number of columns

12

#### Character set

UTF-8

#### Download URL

http://www.faunaeur.org/Data_papers/FaEu_Orthopteroids_2.6.2.zip

#### 

**Data set 2.** 

Column label	Column description
datasetName	The name identifying the data set from which the record was derived (http://rs.tdwg.org/dwc/terms/datasetName).
version	Release version of data set.
versionIssued	Issue data of data set version.
rights	Information about rights held in and over the resource (http://purl.org/dc/terms/rights).
rightsHolder	A person or organization owning or managing rights over the resource (http://purl.org/dc/terms/rightsHolder).
accessRights	Information about who can access the resource or an indication of its security status (http://purl.org/dc/terms/accessRights).
taxonName	The full scientific name of the higher-level taxon.
scientificNameAuthorship	The authorship information for the scientificName formatted according to the conventions of the applicable nomenclaturalCode (http://rs.tdwg.org/dwc/terms/scientificNameAuthorship).
taxonRank	The taxonomic rank of the most specific name in the scientificName (http://rs.tdwg.org/dwc/terms/infraspecificEpithet).
taxonID	An identifier for the set of taxon information (http://rs.tdwg.org/dwc/terms/taxonID).
parentNameUsageID	An identifier for the name usage of the direct parent taxon (in a classification) of the most specific element of the scientificName (http://rs.tdwg.org/dwc/terms/parentNameUsageID).
resourceDescription	An account of the resource, including a data-paper DOI (http://purl.org/dc/terms/description).

## Additional information

For the first compilation of the list of European Orthoptera, Dermoptera, Dictyoptera (Blattaria, Isoptera, Mantodea), Embioptera and Phasmatodea, released at 27 September 2004, the following bibliographic references have mainly been used.

**General taxonomy and faunistics**: Benediktov 1999, Brock 1991, Bukhvalova 1998, Clemente et al. 1989, Clemente et al. 1991, Clemente et al. 1990, Clemente et al. 1999, Coray and Lehmann 1998, Devriese 1996, Gorochov 1987, Gorochov and Marshall 2001, Günther 1995, Harz 1975, Harz and Kaltenbach 1976, Heller 1988, Heller et al. 1998, Jago 1971, Jago 1996, La Greca 1993, La Greca and Lombardo 1983, Massa 1994a, Massa 1994b, Massa 1999, Nadig 1981, Nadig 1991, Nadig 1994, Ragge and Reynolds 1998, Reynolds 1980, Ross 1966, Us and Matvejew 1967

Per country (listing follows the TDWG country codes):

**Andorra (AD)**: Marty 1969, Vahed 1994

**Albania (AL)**: Cejchan 1981, Murraj et al. 1970

**Austria (AT)**: Berg et al. 2000, Schraut 1999

**Bosnia & Herzegovina (BA)**: Adamovic 1956, Miksic 1960, Miksic 1966, Miksic 1967, Miksic 1973, Miksic 1974, Miksic 1978, Miksic 1981

**Belgium (BE**): Decleer 1990, Georges 1984, Naveau 1985

**Bulgaria (BG)**: Bey-Bienko and Peschev 1960, Peshev 1962, Peshev 1964, Peshev 1970, Peshev 1974, Peshev 1975, Peshev and Djingova 1974, Peshev and Maran 1963, Popov et al. 2001

**Switzerland (CH)**: Baur et al. 2000, Thorens and Nadig 1997

**Cyprus (CY)**: Georghiou 1977, Mantovani et al. 1995

**Czech Republic (CZ)**: Chladek et al. 2000, Kocarek et al. 1999

**Germany (DE**): Detzel 2001

**Denmark (DK)**: [Bibr B1202249][Bibr B1202249], Jensen 2002

**Spain (ES)**: Baez 1996, Bland 2001, Bland et al. 1996, Gangwere and Llorente 1992, Garcia et al. 1996, Gomez et al. 1998, Gorochov and Llorente 2001, Herrera Mesa 1982, Llorente and Presa 1997, Olmo-Vidal 1999, Olmo-Vidal and Hernando 2000, Pfau 1996, Pfau and Pfau 1995

**France (FR)**: Defaut 1999, Kruseman 1982, Kruseman 1988, Voisin 2003

**Britain (GB)**: Haes 2003, Hathway et al. 2003, Hawkins 2001, Lee 1998, Marshall and Haes 1990

**Greece (GR)**: Baccetti 1992, Kollaros et al. 1991, Willemse 1984, Willemse 1985

**Hungary (HU)**: Nagy 1997, Nagy et al. 1999, Nagy and Szövenyi 1997

**Italy (IT)**: Baccetti et al. 1995, Failla et al. 1995, Fontana 2001, Fontana and Cussigh 1996, Fontana and La Greca 1999a, Fontana and La Greca 1999b, Fontana and Massa 2000, Fontana and Ode 1999, Fontana et al. 1999, Galvagni 1995, Galvagni 2000, La Greca 1994, La Greca et al. 2000, Schmidt and Herrmann 2000, Schmidt 1996

**Liechtenstein (LI**): Denoth-Hasler 1995

**Luxembourg (LU**): Duijm and Kruseman 1983

**Malta (MT)**: Baccetti 1973, Cassar 1990, Cilia 1975, Harz 1986, Schembri 1984, Schembri and Ebejer 1983, Schembri and Ebejer 1984

**Netherlands (NL)**: Kleukers et al. 1997

**Norway (NO)**: Holst 1986

**Poland (PL)**: Bazyluk and Liana 2000

**Portugal (PT)**: Grosso and Jose 2000, Lange 1990a, Lange 1990b, Lock 1999

**Romania (RO)**: Kis and Vasiliu 1970

**Slovenia (SI)**: Gomboc et al. 2000, Us 1992

**Turkey (TR)**: Karabag 1958, Karabag 1964, Karabag et al. 1971, Naskrecki 1991

**Yugoslavia (YU)**: Adamovic 1975, Cejchan 1981, Pavicevic and Karaman 2001

## Supplementary Material

Supplementary material 1 Fauna Europaea Guidelines for Group Coordinators and Taxonomic Specialists Data type: pdf
File: oo_93845.pdf Yde de Jong, Verner Michelsen, Nicolas Bailly

Supplementary material 2 FaEu Orthopteroid stats Data type: pdf
File: oo_81144.png Yde de Jong

Supplementary material 3 Orthopteroids — Fauna Europaea mapping Data type: xlsx
Brief description: Cross-validation of Fauna Europaea (version 2.6.2) and various Orthopteroid species data sets (version 5.0/5.0), including Orthoptera Species File (http://Orthoptera.SpeciesFile.org), Phasmida Species File (http://Phasmida.SpeciesFile.org), Dermaptera Species File (http://Dermaptera.SpeciesFile.org), and Embioptera Species File (http://Embioptera.SpeciesFile.org). For details on data ownership and correct citation please check the relevant websites.
File: oo_93099.xlsx Yde de Jong and Klaus-Gerhard Heller (Fauna Europaea), Marilyn Beckman, David Eades, and Edward Dewalt (Orthoptera Species File), Paul Brock (Phasmida Species File), Heidi Hopkins (Dermaptera Species File), and Mike Maehr (Embioptera Species File).

## Figures and Tables

**Table 1.** Responsible specialists per family in Orthoptera.

**TAXONOMY**		**EUROPE**	
**FAMILY**	**SPECIALIST(S)**	**DATABASED SPECIES (Fauna Europaea)**	**TOTAL ESTIMATED SPECIES (knowledge-gap)**
Acrididae	Klaus-Gerhard Heller	331	~ 20% more species
Amorphoscelididae	Klaus-Gerhard Heller	1	~ 20% more species
Anisolabididae	Fabian Haas	11	~ 20% more species
Bacillidae	Klaus-Gerhard Heller	7	~ 20% more species
Blaberidae	Horst Bohn	4	~ 20% more species
Blattellidae	Horst Bohn	140	~ 20% more species
Blattidae	Horst Bohn	4	~ 20% more species
Bradyporidae	Klaus-Gerhard Heller	85	~ 20% more species
Conocephalidae	Klaus-Gerhard Heller	11	~ 20% more species
Embiidae	Klaus-Gerhard Heller	9	~ 20% more species
Empusidae	Klaus-Gerhard Heller	5	~ 20% more species
Forficulidae	Fabian Haas	62	~ 20% more species
Gryllidae	Klaus-Gerhard Heller	81	~ 20% more species
Gryllotalpidae	Klaus-Gerhard Heller	14	~ 20% more species
Heteronemiidae	Klaus-Gerhard Heller	6	~ 20% more species
Kalotermitidae	Klaus-Gerhard Heller	4	~ 20% more species
Labiduridae	Fabian Haas	2	~ 20% more species
Mantidae	Klaus-Gerhard Heller	30	~ 20% more species
Meconematidae	Klaus-Gerhard Heller	10	~ 20% more species
Mogoplistidae	Klaus-Gerhard Heller	11	~ 20% more species
Myrmecophilidae	Klaus-Gerhard Heller	9	~ 20% more species
Oligotomidae	Klaus-Gerhard Heller	4	~ 20% more species
Pamphagidae	Klaus-Gerhard Heller	52	~ 20% more species
Phaneropteridae	Klaus-Gerhard Heller	156	~ 20% more species
Phasmatidae	Klaus-Gerhard Heller	4	~ 20% more species
Polyphagidae	Horst Bohn	8	~ 20% more species
Pygidicranidae	Fabian Haas	3	~ 20% more species
Pyrgomorphidae	Klaus-Gerhard Heller	5	~ 20% more species
Rhaphidophoridae	Klaus-Gerhard Heller	49	~ 20% more species
Rhinotermitidae	Klaus-Gerhard Heller	7	~ 20% more species
Spongiphoridae	Fabian Haas	5	~ 20% more species
Termitidae	Horst Bohn	1	~ 20% more species
Tetrigidae	Klaus-Gerhard Heller	12	~ 20% more species
Tettigoniidae	Klaus-Gerhard Heller	222	~ 20% more species
Tridactylidae	Klaus-Gerhard Heller	6	~ 20% more species

**Table 2.** Responsible associated specialists in Orthoptera.

**GROUP or AREA**	**SPECIALIST**
Orthoptera-Saltatoria	Fer Willemse [deceased] — Luc Willemse [follow-up] (see: Willemse and Willemse 2010)
Phasmida	Paul Brock
Corydiidae	Heidi Hopkins
Embioptera	Mike Maehr
Blattaria	George Beccaloni
