# Supplementary material for: Fauna Europaea – Orthopteroid orders
Source: Biodivers Data J. 2016 Jun 29;(4):e8905. doi: 10.3897/BDJ.4.e8905 (PMC5018117; doi:10.3897/BDJ.4.e8905)
Supplement: Supplementary material 1 — Fauna Europaea Guidelines for Group Coordinators and Taxonomic Specialists [file biodiversity_data_journal-4-e8905-s001.pdf]

FAUNA EUROPAEA

The FAUNA EUROPAEA (FaEu) database project – Guidelines for Group Coordinators and Taxonomic Specialists

I. INTRODUCTION

The aim of the 4-year project FAUNA EUROPAEA (hereafter FaEu) is to provide an electronic database focussing on the scientific names and known national /provincial distributions for the ± 100,000 species of terrestrial and limnic metazoans occurring in Europe. It should be emphasised that it is not the scope of the FaEu database to serve as a comprehensive nomenclatorial resource with complete synonymy, indication of type species, type specimens, depositories etc. The backbone of the FaEu database will be the list of accepted scientific names for the different species of animals recognised in Europe linked with information on important synonymy and national / provincial occurrences.

Entries in the FAUNA EUROPAEA database will be linked to external databases containing extended information such as vernacular names in many European languages and conservational status, regionally and nationally. This task will primarily be dealt with by the FaEu project office in Paris and thus of minor concern to the Group Coordinators and Taxonomic Specialists.

Which species-group taxa should be included?

Information on individual species is the main focus of the FaEu database project, but even subspecies may be recorded in some groups (e.g. birds, butterflies and snails), in which they are widely recognised and used. The FaEu database should include all scientifically named and described species (or subspecies) regarded as distinct and belonging to one of the following categories:

- Indigenous, i.e. species naturally belonging to the area, either permanently or as regular (seasonal or occasional) migrants. All species recorded after 1600 should be included, but instances of extinction after that date, regionally or locally, should of course be mentioned under ‘Faunistic Comments’.

- Adventitious, i.e. species that have been introduced into the area; these include
  - organisms that have become naturalised in existing ecosystems
  - invasive organisms that successfully tend to disrupt existing ecosystems
  - synanthropic organisms that can only establish spontaneous, viable populations in 'man-made' habitats

  Remark: Faunistic records of pest species, whether medical, sanitary or agricultural, covered by current quarantine legislations should take utmost care to clearly indicate, in the 'Faunistic Comments' field, if these are based only on past occurrences.

- Cryptogenic, i.e. widespread species not demonstrably indigenous or adventitious in the area

Valid species-group taxa belonging to one of the following categories should be excluded from the FaEu list:

- Domesticated, exotic and other species that do not establish spontaneous, viable populations out of human captivity. Examples: livestock, pets and Zoo animals.
- Exotic imports or casual intruders that for climatic or other reasons cannot establish spontaneous, viable populations.
- Truly fossil or sub fossil species (incl. species that have suffered regional extinction before 1600)

Species found in waters with a low salinity pose a special problem. Those occurring exclusively in brackish seas and adjacent seacoasts should obviously be excluded. However, estuarine species occurring regularly in habitats with fluctuating salinity, e.g. river mouths and semi-closed coastal waters should normally be included.

II. TAXONOMY

Classification – A taxonomic framework is necessary in order to keep track of the thousands of records on species-group taxa to be incorporated into the FaEu database. Well suited for that purpose is the hierarchic classification of scientific names founded by Carolus Linnaeus more than two centuries ago and still 'under construction' by the global community of comparative zoologists. Linnaean classification allows one to choose among hierarchic structures of varying detail.

Higher classification. – For taxa above the family-group (not covered by the provisions of the Zoological Code!) the names and classification adopted by FaEu should follow 'present opinion' among the specialists as far as there exist competing names (synonyms) and competing classifications (different ranks and sequences). For the purposes of the FaEu database the structure of the higher classification is a minor issue, and things might here be kept relatively simple by recognition of very few categories such as phylum - class - order.

APPENDIX 1 provides a discussion proposal on a higher classification of European metazoans based on a variety sources, e.g. the Zoological Record/Biosis and Encyclopedia Britannica. An emended and simplified version of the higher classification given in APPENDIX 1, as approved by leading specialists in the field, will eventually be adopted by the FaEu project.

Lower classification. – As presently defined, this involves the family-, genus- and species-group categories. Three categories only are mandatory: family - genus - species. The Group Coordinators in agreement with the associated Taxonomic Specialists will decide whether to keep the lower classification in the FaEu database simple or complex. Complex hierarchies are for obvious reasons most useful in very species-rich groups. The following are examples of simple and complex classificatory hierarchies for one particular species:

PHYLUM: Arthropoda
- CLASS: Insecta
  - ORDER: Coleoptera
    - FAMILY: Staphylinidae
      - GENUS: *Staphylinus*
        - SPECIES: *Staphylinus erythropterus*

PHYLUM: Arthropoda
- CLASS: Insecta
  - ORDER: Coleoptera
    - SUPERFAMILY: Staphylinoidea
      - FAMILY: Staphylinidae
        - SUBFAMILY: Staphylininae
          - TRIBE: Staphylinini
            - GENUS: *Staphylinus*
              - SPECIES: *Staphylinus erythropterus*

It will also be left to the Group Coordinators and Taxonomic Specialists to decide on the sequence by which taxon names within the -, genus- and species-group categories are entered into the database. It should be emphasised, however, that all taxa of the same rank that belong to the same parent taxon will automatically be put in alphabetical order in the database.

The accepted names for taxa of the family-, genus-, and species-groups are generally established on basis of the priority principle and other provisions of the Zoological Code. The preferred nomenclature in the genus- and species-group categories will

normally follow the taxonomic state-of-the-art from the most recent catalogues, revisions, monographs and nomenclatorial acts. Most specialists do not consider the FaEu database a convenient place for the proposal of new nomenclatorial acts such as new names, new synonymies, new combinations or other changes in the status or application of scientific names. For the same reason, contributors are encouraged to avoid such new proposals in the database and instead to publish them elsewhere. For many taxa there exist a substantial amount of reliable, but unpublished faunistic records. Specialists are encouraged to enter such new faunistic data in order to make the FaEu database the ultimate source on country-based occurrences of animals in Europe. It is recommended that – as a minimum – the depositories for specimens documenting new country records are given in the 'Faunistic comment' field (e.g., '!NL (RMNH)', which means new (!) record for the Netherlands based on material in the National Museum of Natural History, Leiden).

Nomenclature

Treatment of accepted taxon names. – The formation and treatment of scientific names in FaEu context must follow the provisions of the International Code of Zoological Nomenclature, 4th ed. ICZN 1999. Citation of authorship and date for scientific names is – for database purposes – essential in the genus- and species-groups. A few controversial items relating to the formation of species names (= binomina) and authorship are emphasised below:

- Spelling of specific names treated as Latin adjectives: – Opinions have been divided between a spelling conforming with usage, with original spelling, or with generic gender. It should be stressed, however, that the 'generic gender' choice is mandatory according to the Zoological Code and therefore must be followed in the FaEu project. The Diptera volume of 'Checklists of Insects of the British Isles' (Chandler 1998) provides a most useful discussion of the 'generic gender' option and its application.
- Citation of authorship and date for species names: – Most of the accepted specific names are presently combined with a generic name different from the original one. According to the Zoological Code it is mandatory to indicate all such cases by enclosing citations of authorship or authorship and date in parentheses. Notice, however, that parentheses are not released by a subsequent change in subgeneric combination, or by a variant spelling of a generic name.
- The formation of authors' family names: – The following standards should be used in the citation of authorship:
  - Separate prefixes for family names (such as 'da', 'de', 'di', 'von', 'van', 'van der') should always be included. Note: 'De Geer', not 'Degeer' or 'DeGeer', is the correct spelling of the name of the famous Swedish 18th Century entomologist.
  - Spanish, Portuguese and other double names are given in their complete form unless the author uses one name only (e.g., 'Peña' for 'Peña Guzman').
  - For names in non-roman characters, the author's own transliteration is used if known and consistently used (e.g. 'Korneyev', not 'Korneev'; 'Richter', not 'Rikhter').

- The name of an author should be spelled in a consistent way. Thus 'Linné' should not be used as an alternative spelling of 'Linnaeus'.
- Citation of the names of two authors should be separated by '&' (rather than 'and' or 'et'). Authorships involving more than two authors should be fully cited by separating names with commas and – last two names only – a '&'.
- If two or more individuals with identical family names appear as authors of species-group taxa belonging to the same taxon of ordinal or lower rank, these should be distinguished by adding the initials of their first name(s).
- Citation of initials is not mandatory, but if used it is obligated to treat initials of author names before the family/surname.

Synonymy. – 'Synonymous names' in a broad sense, i.e. including variant spellings and misapplications, should normally be kept at a minimum. In well-investigated groups of animals, synonyms may only be needed if

- the application of the accepted name is otherwise equivocal
- a synonym has been in use recently, in Europe or elsewhere

Poorly known groups of animals that have not been taxonomically revised or catalogued for decades may of course require the inclusion of a higher number of synonyms.

Notice that FaEu is not the place for inclusion of *nomina dubia*, i.e. names that cannot be safely associated with any particular taxon. Inclusion of doubtful taxa, because recognised as distinct by some specialists (typically local ones), should be explained in the 'Taxonomic comment' filed.

The FaEu database project distinguishes between 'accepted names' and 'synonymous names'. We use 'accepted' instead of 'valid' because the name in current use may not in all instances be the valid name under the provisions of the Zoological Code. There exist numerous examples in which taxonomists agree that priority rules should be suspended to maintain nomenclatorial usage, but an application to the ICZN is still in preparation or remains to be processed.

Synonymous names are of several kinds. The following refer to genus- and species-group names:

- "True" synonyms, i.e., available names (in the sense of the Zoological Code) that objectively (same type) or subjectively (different type) refer to the same taxon as the accepted name. Unjustified emendations also belong to this category of "true" synonyms. In the FaEu database "true" synonyms should be cited with authorship and date.
- Unavailable names (in the sense of the Zoological Code) such as *nomina nuda* and variant spellings other than unjustified emendations. This category of synonymous names should merely be tagged with 'auct.' instead of full citation of authorship and date.
- Variant applications of available names caused by deviating ideas on contents (lumping/splitting), misidentifications, etc. This category of synonymous names should be tagged with 'auct.' instead of full citation of authorship and date.

Notice that a ‘species name’ and a ‘specific name’ denote two different things. The first refers to the name of a species, i.e. a binominal combination of a generic and a specific name. It is well-known that specific names often appear in the literature in a multitude of different generic combinations. However, it is also worth noticing that different binominal combinations referring to the same species are not synonyms in the sense of the Zoological Code. Therefore, it is outside the scope of the FaEu project to registrate the variety of binominal names that has been in use for a plenitude of species. The only generic combinations with specific names essential for the FaEu database will be:

- The present combination for the accepted specific name
- The original combination for the accepted specific name
- The original combination for any specific name belonging to the above category of "true" synonyms

The reason for asking about the original combination for specific names is two-fold:

- The original binomen serves as a ‘unique identifier’ for a specific name irrespective of its present and future generic placement
- The original combination for an accepted specific name tells us whether or not authorship and date should be cited in parentheses. NB: In situations where the ‘original genus’ is a variant spelling, i.e. an unjustified emendation or a misspelling of the original name, authorship and data should not be cited in parentheses (examples of variant spelling / original spelling: *Pegomyia / Pegomya*; *Otiorrhynchus / Otiorhynchus*).

A nomenclature example on the formation and treatment of scientific names in FaEu – strictly following the provisions of the International Code of Zoological Nomenclature, 4th edition (ICZN 1999) – is included in APPENDIX 2. Main difference with respect to the International Code of Botanical Nomenclature is that – according to the ICZN – binomials are not integrally kept for original combinations ('basionyms') or the different other kinds of synonyms, but stored as uninomials.

References

Chandler P., Ed. (1998) Checklists of insects of the British Isles (New series) Part 1: Diptera. Handbooks for the identification of British insects. Vol. 12, 20 + 234 pp. London: Royal Entomological Society.

ICZN (1999) International Code of Zoological Nomenclature. Fourth Edition. 30 + 306 pp. London: International Trust for Zoological Nomenclature.

The FAUNA EUROPAEA (FaEu) database project – Guidelines for Group Coordinators and Taxonomic Specialists

III. GEOGRAPHIC INFORMATION

IMPORTANT DISCLAIMER

The use of country names in the Fauna Europaea project, both on paper and electronic documents produced and printed, does not express any opinion or judgement concerning the legal status of the countries and other areas named, their sovereignty, their government, their frontiers, their internal administrative subdivisions and boundaries, and their dependencies.

This chapter has been written in order to be as efficient and simple as possible, compared to previous versions, and is recommended to be read first. All what is history, discussions, explanation of choices, non mandatory facilities, difficulties, have been included in the Appendix 3. The latter can be ignored in a first approach. Within the Appendix 3, we have tried to imagine most of the difficulties that can arise, and it should be consulted only for that; some are complex and could be understood only at the moment they arise. At last, it is always possible to contact us for further explanation.

1 Introduction

The FaEu contract states that the species (and subspecies[1]) names should be registered at least at a required country level, meaning political countries.

The two first preliminary reports on geographical issues (Alonso-Zarazaga and Costello, 1996; Bouchet and Legakis, 1997)[2], and the meeting of the Advisory Team on Geographic Information during the General Meeting in Padua (Alonso-Zarazaga and Bailly, 2000) have defined the external boundaries (see Appendix 3, Section 2 for details on the choices): the European area covered by Fauna Europaea is based on what is called "Europe" in TDWG[3] standard (Brummitt, 2001), i.e., geographic Europe up to Ural Mountains including European Turkey excluding Kazakhstan, the Russian Caucasian republics and territories, the independent Transcaucasian republics, and the Asian Turkey: the following additions have been made, the Macaronesian islands (excl. Cape Verde Is.), East Aegean Is., Cyprus, Franz Josef Land and Novaya Zemlya, that are grouped with other higher TDWG units.

In addition, the reports recommended to separate some geographical units from the political country from which they are dependent (mainly archipelagos and islands), and suggested that some extra-FaEu higher levels could be useful for end-users, mainly to evaluate the endemic status.

---

[1] Many discussions at the early beginning of the project have emphasized that subspecies should be taken into consideration, at least for some taxonomic groups, which has been endorsed by the General Meeting in Padua. See section I and II.

[2] During the conception of the project.

[3] Taxonomic Database Working Group (TDWG). See http://www.tdwg.org/

2 The FaEu geopolitical units

All the political countries, even small, must have a separated status and must not be merged with a larger neighbour country (see Appendix 3, Section 3.2 for the list of these areas). Although not strictly stated in the contract, subdivisions of some countries have been established to comply with some (bio)geographical considerations (see Appendix 3, Section 3.3 for the list of these territories).

Only the European part of Russia has been included, and divided according to the TDWG standard that provides five subdivisions (see Appendix 3, Section 3.3.2 for the details of these subdivisions).

69 geopolitical units have been thus considered (see Appendix 3, Table 1).

3 How to enter data

3.1 General Principles

Data can be entered indirectly through an importation step, from a spreadsheet for instance, with the Data Import Tool (DIT), or directly in the database through the Data Entry Tool (DET).

To enter the data in a spreadsheet (or a database), please use the import format of the table given in Appendix 2 that complies with the structure of the database. The raws represent the taxa (considered for faunistic part: species and subspecies), the columns the geopolitical units. There is as many columns as the non-overlapping geopolitical units and extra-FaEu area units that are defined, plus two comment fields. In the cell corresponding to the cross between the taxon raw and the geopolitical unit column, four statuses can be entered:

- Enter “P” to mark "Present": the meaning is « At least one record well documented at least once since year 1600 », except records that are known to be accidental (see chapter I and II).
- Enter “P?” to mark "Doubtful": the meaning can vary, and thus it is recommended that the doubtful records are commented in the *Fine distribution* comment field. The record may be doubtful because: the source is doubtful (reference to a work known not to be at a correct scientific level); possible misidentification; uncertainty of the locality report; uncertainty of the origin of the animal (possible but not verified[4] escape from a zoo, exotic pet abandoned by people, …); species not seen since the year 1600 but not specifically searched in the area (date of last record to be entered in comments if known); …
- Enter “A” to mark “No records”: the meaning is « Not recorded up to now and as far as known by the TSs and the GC ».
- Let the cell empty: see the meaning in the box below.

[4] Note that if these cases are verified, they must not be in FaEu, see sections I and II.

IMPORTANT

At the beginning of the data entry process, by default, all the cells are empty, simply meaning « Not treated », i.e., an empty cell does not mean « No records ».
During the data entry process, this feature provides a working facility of TSs and GCs allowing to see at a glance which units have not been yet treated and to follow the advancement of work.
At the end of the data entry process when it will public, an empty cell means « No data », meaning that neither the TSs nor the GCs have treated the case, or have been able to find the relevant information.

Practical recommendation

During the entry process, and to avoid to enter "A" in all those cells that have been treated and correspond to geopolitical units where taxa have not been yet recorded, which will be the majority, they can be left empty. But at the end of the process, the empty cells remaining must be turned in "A": then think to use automatic replacement available in spreadsheet and equivalent softwares to do it in one single effort. A facility will be provided in the Data Entry Tool as well. Then if really some cells have not been treated at the end of the data entry process, because the information was not available for instance, turn these cells again into empty cells. This will mean « No data », either because there was no time to treat well the case, either the information could not be found. Comments can be added in the *Fine distribution* comment field to explain why the information is not available like "Group not studied in this area, Literature unavailable, etc.", allowing to make differences. This will be very helpful for the final gap analysis.

These general principles are the same to enter data through Internet with the Data Entry Tool; for specific complementary instructions, see the web page http://www.faunaeur.org/det

3.2 Country and subdivision level

As stated above, some of the political countries are replaced by a partition of few subdivisions. These countries as a unique unit are not represented by a column (see Appendix 3, section 4.3.2.1 if the species is reported from the whole country with no indication of the subdivision).

Still, the country level seems to be relevant for some end-users, but we have to be very clear about the meaning of the entries in such cases.

3.3 Example

Let's take the example of France which is subdivided in French mainland (FR-FRA) and Corsica (FR-COR).

rec#	FR-FRA	FR-COR
1	P	A
2	P	
3	A	P
4		P
5	P	P
6	A	A

These records mean that a taxon (species or subspecies) has been recorded in French mainland (rec#1 & 2), in Corsica (rec#3 & 4), in both (i.e., in European political France; rec#5), or has not been recorded from either (i.e., not recorded from European political France; rec#6). Note that rec#1 & 3 are not equivalent to rec#2 & 4 respectively: for rec#1 & 3, the record/no record of the species has been checked for both subdivisions, and consequently marked as "A", whereas for rec#2 & 4, it has not been checked. Therefore, they have not the same meaning.

It is obvious that to know the status of the taxon in a subdivided political country (here France), to create a national list for instance, the relevant requests will merge the subdivision level data.

3.4 Continental level and endemicity

During the meeting of the Advisory Team on Geographic Information in Padua (Alonso-Zarazaga and Bailly, 2000), it has been advocated that there should be a possibility to enter broad distribution data outside Europe, especially to determine the (non)-endemic status. As the extra-limital information is not contractually required, it should be considered as a non-mandatory facility. Two ways are provided:

- 8 broad areas have been established and structured like FaEu geopolitical units (8 columns as *Extra-limital Distribution*); they are intended mainly to be used with status "P", not with "P?" and "A", unless there is a clear need and comment, an empty cell there meaning nothing but "not useful";
- other codes may be used in the first comment field (*Extra-codes*), separated by commas, for units not recognised by FaEu coding system, especially codes from standards like ISO, TDWG, FAO[5], …. When other systems than FaEu one are used, it must be indicated as well in this comment field.

If extra distribution is indicated, one can conclude that the taxon is not endemic to the FaEu area. If the taxon is endemic, but that extra-limital distribution is not entered, it can be stated here: « Endemic for France », « Endemic for FaEu area », etc.

See Appendix 3, Section 4.3.4 for further details.

---

[5] Food and Agricultural Organisation of the United Nations.
See ftp://ftp.fao.org/fi/maps/world_2001.gif

3.5 Fine distribution

In the second comment field (*Fine distribution*), one can enter text, or the complete names of smaller units like islands when there are not in the TDWG-ISO coding system, although it is not the goal of FaEu. Again it is a facility to allow Taxonomic Specialists (TSs) and Group Coordinators (GCs) to enter information that they judge important, or difficult to register elsewhere.

These comment fields *Extra-codes* and *Fine distribution* may be as long as necessary[6], but should be restricted to the minimum information relevant to FaEu.

3.6 Uncertainties

We think it is important to give the possibility to enter the doubt or uncertainty of distributions, by the use of the question mark (P?). But these uncertainties should be commented, and their percentage of the total should be kept as small as possible.

Encoding procedures for uncertainty are explained in Appendix 3, section 4.3.2.2, but it is stressed here that these possibilities can be skipped if they are felt as not necessary by the Taxonomic Specialists (TSs) and Group Coordinators (GCs).

4 Difficulties

How to enter distribution data for subspecies?

See Appendix 3, Section 4.3.1

How to enter data when information is available at a political country but not at a subdivision level?

See Appendix 3, Section 4.3.2.1

How to enter doubtful data?

See Appendix 3, Section 4.3.2.2

Which names and codes for former countries (Czechoslovakia, former Yugoslavia, Eastern Germany, etc.) to be used only in comment fields?

See Appendix 3, Section 4.3.2.3, Table 2

How to enter infra-country data?

See Appendix 3, Section 4.3.3

How to indicate endemic status?

See Appendix 3, Section 4.3.4

[6] In spreadsheets and some type of fields in DBMS (DataBase Management System), the length of a cell may be restricted to 255 characters. Add as many field as necessary with ordered title like *Fine distribution 2*, *Fine distribution 3*, etc.

For any further discussion or information on geography in FaEu, please do not hesitate to contact the FaEu Paris Office (http://www.mnhn.fr/iga/membres/NB/FaEu, faunaeur@mnhn.fr), as well as the main FaEu site where documents are available for download (http://www.faunaeur.org/partners/partnerweb/alldocuments/alldocs1.html).

References

Alonso-Zarazaga M.A. & Bailly N. (2000). Geography. Report 3 (June 2000). Fauna Europaea.

Alonso-Zarazaga M.A. & Costello M.J. (1996). Geography. Report 1 (August 1996). Fauna Europaea.

Anonymous (1997-1999). Codes for the representation of names of countries and their subdivisions. ISO 3166 standard. Part 1: Country codes (1997), 58 p.; Part 2: Country subdivision code (1998), 105 p.; Part 3: Code for formerly used names of countries (1999), 12 p. Paris La Défense: AFNOR.

Bouchet P. & Legakis A. (1997). Faunal boundaries of the "Fauna Europaea" project. Report 2 (May 1997). Fauna Europaea.

Brummitt R.K. (2001). World geographical scheme for recording plant distributions. Edition 2. Plant Taxonomic Database Standards n°2. International Working Group on Taxonomic Databases for Plant Sciences (TDWG). Pittsburgh: Hunt Institute for Botanical Documentation, Carnegie Mellon University. xv+137 p.

APPENDIX 1. Higher classification (ranked above family-group level) **of the terrestrial and limnic metazoans of Europe.**

ID	Phylum	Subphylum	Class	Subclass	Superorder	Order	Suborder
1	Porifera<br>(sponges)	Cellularia	Demospongiae<br>(siliceous sponges)	Ceractinomorpha		Haplosclerida	
2	Cnidaria<br>(cnidarians)<br>= Coelenterata		Hydrozoa			Hydroida	Hydrina<br>= Anthomedusae
							Limnohydrina<br>= Limnomedusae
							Athecata
3	Rotifera<br>(rotifers, wheel animalcules)<br>= Rotatoria		Bdelloidea				
			Monogononta				
4	Acanthocephala<br>(spiny-headed worms)		Archiacanthocephala				
			Eoacanthocephala			Gyracanthocephala?	
						Neoechinorhynchida	
			Palaeacanthocephala			Echinorhynchida	
						Polymorphida	
5	Gastrotricha<br>(gastrotrichs);<br>188 spp.		Chaetonotida			Chaetonotoidea	Multitubulatini
							Paucitubulatini
6	Nematoda<br>(nematodes, roundworms, eelworms)<br>= Nemata		Adenophorea			Enoplida	
						Trefusiida	
						Dorylaimida	Dorylaimina
							Bathyodontina
							Mononchina
						Isolaimida	
						Monhysterida	
						Chromadorida	
						Stichosomida	
			Rhabditia<br>= Secernentea			Rhabditida	
						Strongylida	
			Spiruria			Ascaridida	
						Spirurida	
						Diplogasterida	
						Tylenchida	
7	Nematomorpha<br>(horsehair worms, gordian worms)		Gordioida			Gordiida	
8	Platyhelminthes<br>(flatworms)		Turbellaria<br>(turbellarians)			Catenulida	
						Macrostomida	
						Lecithoepithelia	
						Prolecithophora	
						Proseriata	
						Seriata	Tricladida<br>(planarians)
						Rhabdocoela	
			Trematoda (flukes)	Digenea		Gasterostomata	
						Prosostomata	
				Monogenea			
				Aspidogastrea<br>= Aspidobothria<br>= Aspidocotylea			
			Cestoda (tapeworms)	Cestodaria		Amphilinidea	
				Eucestoda		Spathebothriidea	
						Caryophyllidea	
						Tetraphyllidea	
						Pseudophyllidea	
						Proteocephalidea	
						Tetrabothriidea	
						Cyclophyllidea	
9	Nemertini<br>(ribbon worms)<br>= Nemertea<br>= Rhynchocoela		Enopla			Hoplonemertini	

10	Entoprocta = Endoprocta		Kamptozoa			Coloniales	Stolonata
11	Bryozoa (moss animals) = Ectoprocta = Polyzoa		Gymnolaemata	Cheilostomata			
				Ctenostomata			
			Phylactolaemata	Cyclostomata = Tubuliporata			
12	Mollusca (mollusks)		Gastropoda (snails)	Neritimorpha = Neritopsina		‘Neritoidea’	
				Caenogastropoda		Architaenioglossa	
						‘Cerithioidea’	
				Heterobranchia		‘Valvatoidea’	
						Pulmonata	Basommatophora
							Stylommatophora
			Bivalvia (bivalves, clams, mussels) = Pelecypoda = Lamellibranchia	Palaeoheterodonta		Unionoida (naiads, pearly freshwater muscles)	
				Heterodonta		Veneroida	
13	Annelida	Polychaeta (bristleworms)				Sabellida (fanworms, feather dusters)	
						Archiannelida = Nerillida	
		Clitellata (clitellate worms)		Oligochaeta		Haplotaxida	Haplotaxina (groundwater worms)
							Tubificina (sludge worms)
							Lumbricina (earthworms)
				Branchiobdellida		Branchiobdellea	
				Acanthobdellea		Acanthobdellida	
				Hirudinea (leeches)		Rhynchobdellida = Rhynchobdellae	
						Arhynchobdellida = Arhynchobdellae	Hirudiniformes
							Erpobdelliformes
			Aphanoneura				
14	Tardigrada (water bears)		Heterotardigrada			Arthrotardigrada	
						Echiniscoidea	
			Eutardigrada			Parachela	
						Apochela	
15	Arthropoda (arthropods)	Chelicerata	Arachnida (arachnids)			Scorpiones (scorpions) = Scorpionida	
						Palpigradi (micro whipscorpions)	
						Solifugae (sunspiders or wind scorpions) = Solpugida	
						Amblypygi (whipscorpions)	
						Uropygi (whipscorpions) = Thelyphonida	
						Opiliones (harvestmen)	
						Pseudoscorpiones (false scorpions or book scorpions) = Pseudoscorpionida	
						Araneae (spiders) = Araneida	Orthognatha (mygalomorph spiders)
							Lapidognatha (araneomorph spiders)
				Acari (mites) = Acarina	Opilioacarida	Notostigmata	
					Anactinotrichida = Parasitiformes	Ixodida (ticks)	
						Holothyrida	
						Gamasida = Mesostigmata	
					Actinotrichida	Acaridida	Acaridina

					= Acariformes	=Astigmata		
							Psoroptidina	
						Actinedida<br>= Prostigmata<br>= Trombidiformes		
						Oribatida<br>= Cryptostigmata<br>= Oribatei		
						Tarsonemida		
		Crustacea (crustaceans)	Branchiopoda			Anostraca		
						Notostraca		
						Spinicaudata		
						Cladocera		
			Maxillopoda					
				Ostracoda (mussel- or seed-shrimps)		Podocopida	Podocopina	
				Branchiura		Arguloida (fish lice)		
				Copepoda		Calanoida		
						Cyclopoida	Gnathostoma	
						Gelyelloida		
						Harpacticoida		
						Poecilostomatoida		
						Siphonostomatoida		
			Malacostraca	Eumalacostraca	Syncarida	Bathynellacea		
					Peracarida	Mysidacea (opossum shrimps)		
						Isopoda	Asellota	
							Microcerberidea	
							Oniscidea (wood lice)	
							Flabellifera	
						Amphipoda (beach hoppers, well shrimps)	Gammaridea	
							Ingolfiellidea	
					Eucarida	Decapoda (shrimps, prawns, lobsters, crayfish, crabs)		
			Pentastomida			Porocephalida		
					Pancarida	Thermosbaenacea		
		Myriapoda (myriapods)	Chilopoda (centipedes)			Scutigeromorpha		
						Lithobiomorpha		
						Scolopendromorpha		
						Geophilomorpha		
			Symphyla (symphylans)					
			Diplopoda (millipedes)			Polyxenida		
						Glomerida		
						Platydesmida		
						Polyzoniida		
						Callipodida		
						Chordeumatida		
						Polydesmida		
						Julida		
			Pauropoda (pauropods)					
		Hexapoda	Entognatha			Diplura		
					Ellipura	Collembola (springtails)		
						Protura		
			Insecta (insecs)<br>= Ectognatha			Archaeognatha<br>= Microcoryphia (bristletails)		
						Zygentoma (silverfish)		
				Pterygota		Ephemeroptera (mayflies)		
						Odonata (dragonflies, damselflies)	Zygoptera	
							Anisoptera	
						Plecoptera (stoneflies)		
						Dictyoptera	Mantodea (praying mantids)	
							Blattodea	

							(cockroaches)
							Isoptera (termites)
						Orthoptera (grasshoppers)	Ensifera (crickets, katydids, long-horned grasshoppers)
							Caelifera (locusts, short-horned grasshoppers
						Phasmatodea (stick-insects)	
						Dermaptera (earwigs)	
						Embioptera (webspinners, footspinners)	
						Psocodea (psocids)	Psocoptera (barklice, booklice)
							Phthiraptera (lice)
						Thysanoptera (thrips)	
						Hemiptera (hemipterans)	Sternorrhyncha (psyllids, whiteflies, aphids, coccids, mealybugs, scale insects)
							Auchenorrhyncha (cicadas, leaf-hoppers)
							Heteroptera (bugs)
					Endopterygota = Holometabola	Megaloptera (alderflies)	
						Raphidioptera (snakeflies)	
						Neuroptera (lacewings) = Planipennia	
						Coleoptera (beetles)	Archostemata
							Myxophaga
							Adephaga
							Polyphaga
						Hymenoptera (sawflies, wasps, bees, ants)	
						Trichoptera (caddisflies)	
						Lepidoptera (moths, butterflies)	
						Mecoptera (scorpionflies)	
						Siphonaptera (fleas)	
						Diptera (true flies)	
						Strepsiptera (strepsipterans)	
16	Vertebrata (vertebrates)	Agnatha (jawless fishes)	Cephalaspidomorphi			Petromyzontiformes (lampreys)	
			Osteichthyes (bony fishes)			Acipenseriformes (sturgeons)	
				Teleostei		Anguilliformes	Anguilloidei (freshwater eels)
						Clupeiformes (herrings)	
						Cypriniformes (carps)	
						Siluriformes (catfishes)	
						Salmoniformes	Esocoidei (pikes, mudminnows)
							Salmonoidea (salmon, trout)
						Gadiformes (cods)	
						Cyprinodontiformes	
						Atheriniformes	
						Gasterosteiformes (sticklebacks)	
						Syngnathiformes (pipefishes)	
						Scorpaeniformes	

Nomenclature Example

Family ANTHOMYIIDAE

***Acklandia* Hennig 1976**
= *Phorbia* auct.

***Acklandia servadeii* (Séguy 1933)** [*Hylemyia*]
Distribution: CZ, DE, HU, IT-ITA, RO, SK. Extralimital: ASI.

***Acridomyia* Stackelberg 1929**

***Acridomyia sacharovi* Stackelberg 1929** [*Acridomyia*]
Distribution: FR-FRA, RU-RUS. Extralimital: ASI.

***Acyglossa* Rondani 1866**

***Acyglossa atramentaria* (Meigen 1826)** [*Anthomyia*]
Distribution: AT, DE, FR-FRA, HR, HU, IT-ITA, RO, SK. Extralimital: ASI.

***Acyglossa pollinosa* Villeneuve 1908** [*Acyglossa*]
Distribution: CH, FR-FRA, IT-ITA. Extralimital:

***Adia* Robineau-Desvoidy 1830**
= *Paregle* auct.
= *Scategle* Fan 1982

***Adia cinerella* (Fallén 1825)** [*Musca*]
Distribution: AC, AL, AT, BG, CH, CY, CZ, DE, DK-DEN, EE, ES-BAL, ES-CNY, ES-SPA, FI, FR-COR, FR-FRA, GB-GRB, GR-DOD, GR-GRC, GR-KRI, HR, HU, IT-ITA, IT-SI, LV, MD, NL, NO-NOR, PL, PT-AZO, PT-MDR, PT-POR, RO, RU-RUN, RU-RUS, SE, SK, UA, YU. Extralimital: ASI, NEA, ORI.

***Adia grisella* (Rondani 1871)** [*Chortophila*]
Distribution: CH, DE, IT-ITA. Extralimital: ASI.

***Alliopsis* Schnabl & Dziedzicki 1911**
= *Analliopsis* Fan 1983
= *Circia* Malloch 1929
= *Paraprosalpia* Villeneuve 1922
= *Pseudochirosia* Ringdahl 1928
= *Sinoprosa* Qian & Fan 1981

***Alliopsis albipennis* (Ringdahl 1928)** [*Pseudochirosia*]
Distribution: GB-GRB, NO-NOR, RU-RUN, SE. Extralimital: NEA.

***Alliopsis aldrichi* (Ringdahl 1934)** [*Prosalpia*]
Distribution: FI, RU-RUN, SE. Extralimital: ASI, NEA.

***Alliopsis atronitens* (Strobl 1893)** [*Anthomyia*]
Distribution: AT, FI, GB-GRB, IT-ITA, NO-NOR, RU-RUN, SE. Extralimital: ASI.

***Alliopsis benanderi* (Ringdahl 1926)** [*Hylemyia*]
Distribution: FI, NO-NOR, RU-RUN, SE. Extralimital: NEA.

***Alliopsis billbergi* (Zetterstedt 1838)** [*Anthomyza*]
Distribution: AT, CH, CZ, DE, DK-DEN, EE, ES-SPA, FI, FR-FRA, GB-GRB, NL, NO-NOR, PL, RU-RUN, SE, SK. Extralimital: ASI.

***Alliopsis brunneigena* (Schnabl 1915)** [*Prosalpia*]
= *incisa* Ringdahl 1926 [*Prosalpia*]
Distribution: CZ, EE, FI, NO-NOR, RU-RUN?, SE. Extralimital: NEA.

***Alliopsis conifrons* (Zetterstedt 1845)** [*Aricia*]
= *similaris* d'Assis Fonseca, 1966 [*Paraprosalpia*][1]
Distribution: AT, CH, CZ, DE, DK-FOR, ES-SPA, FI, GB-GRB, NO-NOR, PL, RU-RUN, SE. Extralimital: ASI, NEA.

***Alliopsis denticauda* (Zetterstedt 1838)** [*Anthomyza*]
Distribution: FI, NO-NOR, RU-RUN, SE. Extralimital: ASI, NEA.

***Alliopsis dentiventris* (Ringdahl 1918)** [*Prosalpia*]
Distribution: DE, DK-DEN, FI, NO-NOR, RU-RUN, SE. Extralimital: NEA.

***Alliopsis fractiseta* (Stein 1908)** [*Chirosia*]
Distribution: FI, NO-NOR, RU-RUN, SE. Extralimital: NEA.

***Alliopsis freyi* (Ringdahl 1932)** [*Hylemyia*]
Distribution: FI, NO-NOR, RU-RUN, SE. Extralimital:

***Alliopsis glacialis* (Zetterstedt 1845)** [*Aricia*]
Distribution: AT, CH, FI, FR-FRA, IT-ITA, NO-NOR, RU-RUN, SE. Extralimital: ASI, NEA.

***Alliopsis laminata* (Zetterstedt 1838)** [*Anthomyza*]
Distribution: SE. Extralimital: NEA.

***Alliopsis longiceps* (Ringdahl 1935)** [*Hylemyia*]
= *austriaca* Hennig 1976 [*Paraprosalpia*][2]
= *sitiens* Collin 1943 [*Prosalpia*][3]
Distribution: AT, GB-GRB, RU-RUN. Extralimital:

***Alliopsis longipennis* (Ringdahl 1918)** [*Chortophila*]
Distribution: FI?, NO-NOR, SE. Extralimital: NEA.

---

[1] Males of Alliopsis conifrons exhibit considerable geographic variation with respect to dusting pattern, profile of head, frontal width and setation of mesotibiae and metafemora. The original description of males of P. similaris from Scotland lists features that readily distinguishes it from Fennoscandian males of A. conifrons. However, other geographical variants are found in the Central European mountains and in the Spanish Pyrenees. I conclude therefore that Paraprosalpia similaris d'Assis Fonseca is best treated as a junior synonym (SYN. N:) of Alliopsis conifrons (Zetterstedt).

[2] Males of Alliopsis conifrons exhibit considerable geographic variation with respect to dusting pattern, profile of head, frontal width and setation of mesotibiae and metafemora. The original description of males of P. similaris from Scotland lists features that readily distinguishes it from Fennoscandian males of A. conifrons. However, other geographical variants are found in the Central European mountains and in the Spanish Pyrenees. I conclude therefore that Paraprosalpia similaris d'Assis Fonseca is best treated as a junior synonym (SYN. N:) of Alliopsis conifrons (Zetterstedt).

[3] See previous footnote for details on synonymy.

***Alliopsis maculifrons* (Zetterstedt 1838)** [*Anthomyza*]
= *silvestris* auct.
Distribution: FI, NO-NOR, SE. Extralimital:

***Alliopsis moerens* (Zetterstedt 1838)** [*Anthomyza*]
Distribution: FI, NO-NOR, RU-RUN, SE. Extralimital: ASI, NEA.

***Alliopsis obesa* Malloch 1919** [*Alliopsis*]
= *imprompta* Huckett 1965 [*Alliopsis*]
Distribution: NO-NOR, SE. Extralimital: NEA.

***Alliopsis pilitarsis* (Stein 1900)** [*Prosalpia*]
Distribution: AT, CH, DE, ES-SPA, GB-GRB, PL, RO, SE, SK. Extralimital: ASI, NEA.

***Alliopsis rambolitensis* (Villeneuve 1922)** [*Prosalpia*]
Distribution: FR-FRA, GR-GRC, GR-KRI. Extralimital:

***Alliopsis sepiella* (Zetterstedt 1845)** [*Aricia*]
Distribution: AT, CZ, DE, DK-DEN, ES-SPA, FI, GB-GRB, IS, NO-NOR, PL, SE, SK. Extralimital: ASI, NEA.

***Alliopsis silvatica* (Suwa 1974)** [*Paraprosalpia*]
Distribution: RU-RUN. Extralimital: ASI.

***Alliopsis silvestris* (Fallén 1824)** [*Musca*]
Distribution: AT, BG, CH, CZ, DE, DK-DEN, EE, ES-SPA, FI, FR-FRA, GB-GRB, HU, NL, NO-NOR, PL, RU-RUN, SE, SK. Extralimital: ASI, NEA.

***Alliopsis teriolensis* (Pokorny 1893)** [*Prosalpia*]
= *borealis* Stein 1916 [*Prosalpia*]
Distribution: AT, CH, ES-SPA, FI, IT-ITA, NO-NOR, RU-RUN, SE. Extralimital: NEA.

Nomenclature diagrams

A help example on nomenclature can be found at:

http://www.faunaeur.org/Guide/HelpExample.html

						Perciformes (perches)	
			Amphibia (amphibians)	Lissamphibia		Caudata (salamanders)<br>= Urodela	
						Anura (frogs, toads)	
			Reptilia (reptiles)	Anapsida		Testudines (turtles, tortoises)<br>= Chelonia	
				Lepidosauria		Squamata	Sauria (lizards)
							Serpentes (snakes)
							Amphisbaenia (worm lizards)
			Aves (birds)	Neornithes	Neognathae	Gaviiformes (divers, loons)	
						Podicipediformes (grebes)	
						Procellariiformes (petrels, shearwaters)	
						Pelecaniformes (pelicans, gannets, cormorants)	
						Ciconiiformes (herons, storks, ibises)	
						Phoenicopteriformes (flamingos)	
						Anseriformes (ducks, geese, swans)	
						Accipitriformes (vultures, eagles, buzzards, hawks)	
						Falconiformes (falcons)	
						Galliformes (fowl, game birds)	
						Gruiformes (cranes, bustards, gallinules)	
						Charadriiformes (gulls, shore birds)	
						Pteroclidiformes (sandgrouse)	
						Columbiformes (doves, pigeons)	
						Cuculiformes (cuckoos)	
						Strigiformes (owls)	
						Caprimulgiformes (nightjars)	
						Apodiformes (swifts)	
						Coraciiformes (kingfishers, bee eaters, rollers, hoopoes)	
						Piciformes (woodpeckers)	
						Passeriformes (passerines, perching birds)	
			Mammalia (mammals)	Theria (viviparous mammals)	Eutheria [infraclass] (placentals)	Insectivora hedgehogs, moles, shrews)	
						Chiroptera (bats)	
						Lagomorpha (picas, hares, rabbits)	
						Rodentia (rodents)	Sciuromorpha (squirrels, beavers)
							Myomorpha (rats, mice, dormice, voles, hamsters, lemmings
							Hystricomorpha (porcupines, guinea-pigs)
						Carnivora (carnivores)	Caniformia (dogs, foxes, bears, weasels, otters
							Feliformia (cats,

							civets, hyenas?)
						Artiodactyla (even-toed ungulates	Suiformes (pigs)
							Tylopoda (camels?)
							Ruminantia (ruminants: deer, cattle, goats, sheep)
						Perissodactyla (odd-toed ungulates: horses)	
						Primates (primates)	Haplorhini (monkeys)

1 Introduction

This appendix deals with the choice of the external geographical boundaries of the FaEu area, the definition the internal geopolitical units that are mandatory to consider and those that are possible to use, and the way to enter the data (coding and fields) in the import format, and it details the difficulties that may be encountered. Although the political country is the required level, the two first initial reports on geographical issues (Alonso-Zarazaga and Costello, 1996; Bouchet and Legakis, 1997), and the meeting of the Advisory Team on Geographic Information during the General Meeting in Padua (Alonso-Zarazaga and Bailly, 2000) have recommended to separate some geographical units from the political country from which they are dependent (mainly archipelagos and islands). It has been suggested also that some higher level could be useful for end-users as well, mainly to evaluate the endemic status.

In order to provide both facilities (from sub- to supra-country levels), the FaEu geographical system will follow the ISO 3166[7]-TDWG[8] standards.

Basically, the country level (political level) will follow the ISO 3166-1 standard for the names and codes because the country level is of first interest for FaEu. The TDWG standard document provides the equivalence with ISO.

The subdivision level (geopolitical level) is a compatible adaptation (see Table 1) of the TDWG standard, 2nd edition[9] (Brummitt, 2001). The ISO 3166-2 standard concerns also the subdivisions, but the coding system is heterogeneous from one country to another and the more homogeneous TDWG system has been preferred. The adaptation concerns the fact that in FaEu, we first want to gather data from the subdivisions recognised for only one political country, while the TDWG system is more based on geographical gatherings. For example, the Channel Is. are under a broad unit called "France" (here not the political country), code FRA, which groups French mainland, Monaco and Channel Is. So the adapted codes provides a link between ISO and TDWG standards, allowing both gatherings at country and biogeopolitical levels. Names are given in English when they exist in TDWG (mainly countries and well known areas like archipelagos and large islands) and in native language from ISO (mainly for administrative subdivisions when no English name is obvious or frequently used).

For the outside FaEu area world level, two systems may be used indifferently, according to their ability to closely represent the distribution: the FaEu propositions (strict biogeographical level, see Table 3) and/or the TDWG standard (biogeopolitical level, see Table 4). If a (sub)species is recorded in at least one European country, and is not registered outside FaEu area within one of these two systems, the taxon may be considered as endemic for the FaEu area.

---

[7] Note that this standard of the International Organization for Standardization (ISO) is composed of three parts: 3166-1 for the names and codes of the countries; 3166-2 for the names and codes for their subdivisions; 3166-3 for the names and codes for former countries. See http://www.iso.ch/

[8] Taxonomic Database Working Group (TDWG).
See http://www.bgbm.fu-berlin.de/TDWG/geo/default.htm

[9] The version 2 has been recently published, August 2001. Changes had already been anticipated before this publication to provide the FaEu code in time, and are compliant with the geographic system in Euro+Med PlantBase, also based on the same standards.

2 The external boundaries of the project

Initial reports and additional discussion (summarised in Padua) have pointed out that there is no objective automatic criteria in order to include or exclude specific countries, or part of countries. Thus, the geographical limits have been selected[10] with a weighted combination of six criteria sets.

(a) political criteria;
(b) scientific criteria;
(c) administrative limits between subdivisions;
(d) limits for the European Environment Agency;
(e) existing standards;
(f) dependencies and overseas territories.

2.1 General definition

The European area covered by Fauna Europaea is based on what is called "Europe" in TDWG standard (Brummitt, 2001), i.e. geographic Europe up to Ural Mountains including European Turkey excluding Kazakhstan[11], the Russian Caucasian republics and territories, the independent Transcaucasian republics, and the Asian Turkey: the following additions have been made, the Macaronesian islands (excl. Cape Verde Is.), East Aegean Is., Cyprus, Franz Josef Land and Novaya Zemlya.

2.2 Details of the criteria

(a) Political criteria, i.e., the end-product should be attractive and useful to funding agencies and biodiversity management users. In this context, the concept of "Minimal Europe" includes the terrestrial sovereign territory of all the member states of the EU, including Madeira, the Azores, the Selvagens Is. and the Canary Is.

(b) Scientific criteria, i.e., comprehensive inclusion of whole biogeographical regions or sub-regions. In this context, the consensus is that "Minimal Europe" includes all the countries West of the Ural Mountains, including the New Accessing States (NAS), former USSR republics, and current Russian republics, oblasts, and okrugs.

In this biogeographical context, Alonso-Zarazaga and Costello (1996) also investigated an extension of a "Maximal Europe" to non-European countries of the Western Palearctic (North Africa, Middle East). From a scientific point of view, the merit of this approach is a global inclusion of the Western Palearctic within FaEu. But, conversely, their higher biodiversity should significantly increase the total number of the taxa to be registered, and most of these countries had no direct political agreement to be part of a RTD-FP5 project. Thus, Bouchet and Legakis (1997) strongly advocated for the "Minimal Europe", which has been confirmed by Alonso-Zarazaga and Bailly (2000).

[10] These decisions are obviously arbitrary (especially the Asian Turkey-Caucasus region), but are no more negotiable, the discussion in Padua having been the final one to allow the beginning of the work. People who would like to work on non-included areas are strongly invited to present other projects in the FP6 (6th European Framework Programme for Research and Technological Development -FP6), especially in the INCO programme, to extend the FaEu coverage to Caucasus region, for instance. Note that since the version 3 of the guidelines, a proposal has been accepted regarding the participation of NAS countries (Newly Accessing States) to Fauna Europaea, which includes Cyprus, Malta, East European countries, but not the former USSR republics, and which does not extend the area covered by Fauna Europaea.

[11] Note that West Kazakhstan has been transferred from South European Russia in the 1st edition of the TDWG standard to Middle Asia in the 2nd edition (Brummitt, 2001).

(c) Administrative limits between subdivisions inside the countries which are not totally covered by FaEu (mainly Russia).

(d) Existing boundaries established by the European Environment Agency in the framework of the extension of the directive Natura 2000 towards an Emerald Network of Areas of Special Conservation interest for the Palearctic region.

(e) Existing boundaries used by European faunas, floras or monographs, or in international standards (as the ones by Taxonomic Database Working Group, TDWG).

(f) Dependencies clearly belonging to the mainland of another continent are excluded (British, Dutch and French overseas, Spanish north African territories, etc.).

2.3 Details of boundaries

2.3.1 Northern boundaries (from west to east)

2.3.1.1 Main criteria

- Inclusion of the Northern Atlantic islands

2.3.1.2 Territories included at the boundaries

- Svalbard
- Franz Josef Land (Ushakova and Vize Is. are excluded since they are not part of Arkhangel'skaya oblast' like Franz Josef Land)
- The northern coast of continental Scandinavia
- The northern coast of continental Russia (Arkhangel'skaya oblast', incl. Nenetskiy Avtonomnyy Okrug)
- Novaya Zemlya

2.3.2 Eastern boundaries (from north to south)

2.3.2.1 Main criteria

- The administrative limits following most closely the mountain line of the Ural Mountains
- The exclusion of Kazakhstan

2.3.2.2 Territories included at the boundaries

- Novaya Zemlya
- The eastern borders of Arkhangel'skaya oblast' (incl. Nenetskiy Avtonomnyy Okrug), Komi Respublika, Permskaya Oblast' (incl. Komi-Permyatskiy Avtonomnyy Okrug) and Bashkortostan Respublika (Sverdlovskaya Oblast'[12] and Chelyabinskaya Oblast' are excluded)
- The Russia - Kazakhstan border
- Astrakhanskaya Oblast' and Kalmykiya Respublika Caspian coasts (Dagestan Respublika is excluded)

2.3.3 South-eastern boundaries (from east to west then south)

2.3.3.1 Main criteria

- Exclusion of all the Caucasus region (thus excluding both the Russian Caucasian republics and territories, the independent Transcaucasian republics and the Asian Turkey)
- North and west coast of the Black Sea from Rostovskaya Oblast' to European Turkey
- Inclusion of Cyprus

---

[12] Yekaterinburg is now the official name of the Oblast center city, which is different from other Oblasts where the center city gives the name to the Oblast.

2.3.3.2 Territories included at the boundaries

- The southern borders of the Kalmykiya Respublika and the Rostovskaya Oblast' (Stavropol'skiy Kray and Krasnodarskiy Kray are excluded).
- Azov Sea coast of Rostovskaya Oblast', Azov Sea coast and Black Sea coasts of Ukraine, Black Sea coast of Romania and Bulgaria, Black Sea and Marmara Sea coasts of European Turkey (i.e. west of Bosporus Strait, of Marmara Sea, of Dardanelles Strait; the Imroz I. - Gökçeada - is included; the islands in the Marmara Sea are excluded)
- The Turkey-Greece border (i.e. all the Greek islands in Aegean Sea are included even if they are closer to Asian Turkey)
- Cyprus and surrounding islets

2.3.3.3 Comment on eastern and south-eastern Caucasian boundaries

A modification is proposed on map 2 in Alonso-Zarazaga and Costello (1996) presented in Padua, because at that time, they followed Tutin *et al.* (1964) who had chosen main river lines as limits to keep all the watersheds, both on the Caspian and Azov seas coasts. We have excluded the Caucasian Mountains and Plateau completely, just like TDWG standard. Following strictly the administrative borders, the Dagestan Respublika (with the Caspian watersheds), the Stavropol'skiy Kray (with the Caucasian Plateau) and the Krasnodarskiy Kray (with the Azov watersheds) are excluded.

2.3.4 Southern Boundaries (from east to west)

2.3.4.1 Main criteria

- Inclusion of Cyprus
- Inclusion of all the European Mediterranean islands
- Inclusion of Macaronesian islands (excl. Cap Verde Is.)

2.3.4.2 Territories included at the boundaries

- Cyprus and surrounding islets
- Crete and surrounding islets
- Southern coast of continental Europe from Greece to Gibraltar and Portugal
- Sicily
- Malta and surrounding islets
- Small Italian islands: Lampedusa, Lampione, Linosa (= Pelagie Is. all three together), Pantelleria
- Sardinia
- Balearic Is.
- Alboran I.
- Canary Is.

2.3.5 Western boundaries (from south to north)

2.3.5.1 Main criteria

- Inclusion of the Macaronesian Is. (excl. Cap Verde Is.)
- Exclusion of Greenland

2.3.5.2 Territories included at the boundaries

- Canary Is.
- Selvagens Is.
- Madeira
- Azores
- The western coast of continental Europe from Portugal to Norway

- British Is. (Scilly Is. and Rockall are included)
- Faroe Is.
- Iceland
- Jan Mayen
- Svalbard

3 The FaEu geopolitical units

3.1 Introduction

All the political countries, even small, must have a separated status and must not be merged with a larger neighbour country. Although not strictly stated in the contract, subdivisions of some countries have been established to comply with some (bio)geographical considerations, mainly to identify some large islands and archipelagos. Also, Russia is a large country and only the European part has been included, and divided according to the TDWG standard that provides five subdivisions.

69 geopolitical units have been thus considered (see Table 1).

Other ecological or biogeographical subdivision systems of the European mainland could have been envisaged, but are not currently used in FaEu, although we strongly recommend a more thorough study at a later stage of the project or in a following project like ENBI, in collaboration with the European Environment Agency, and other European projects like Euro+Med PlantBase.

3.2 The small political units

- Andorra
- Liechtenstein
- Monaco
- San Marino
- Vatican City State

3.3 Separated territories, archipelagos and islands

For example, Denmark = Danish mainland + Faroe Is.

3.3.1 Others than Russia

- Danish mainland (incl. Bornholm I.)
  - Faroe Is.
- French mainland (incl. all the near-shore islands except Channel Is.)
  - Corsica and surrounding islets
- Great Britain I. (incl. Scotland, Shetlands, Orkneys, Hebrides, England, Man I., Wales)
  - Channel Is. (Jersey, Guernsey, Alderney)
  - Gibraltar
  - Northern Ireland
- Greek mainland (incl. all other islands, Andikíthira I., Evvia I., Ionian Is., Samothráki I., Northern Sporades Is., Thásos I.)
  - Crete (incl. small adjacent islands like Gávdhos)
  - Cyclades Is.
  - Dodecanese Is.
  - North Aegean Is.

- Italian mainland (incl. all other islands)
  - Sardinia
  - Sicily and adjacent Italian islands (Lipari Is., Ustica I., Egadi Is., Pantelleria I., Pelagie Is. = Lampedusa, Lampione and Linosa)
- Norwegian mainland (incl. all other islands)
  - Svalbard, Bear I. and Jan Mayen
- Portuguese mainland
  - Azores
  - Madeira
  - Selvagens Is.
- Spanish mainland (incl. Alboran I.)
  - Balearic Is.
  - Canary Is.
- European Turkey (incl. Imroz I., but not the islands in the Sea of Marmara)

Some other islands could have been taken into consideration as well, but no objective selection criteria does exist at the moment. We suspect that the selection of given territories depends on each taxonomic group, therefore the system is kept as simple as possible. We consider that the check-list management of these small territories should remain the responsibility of the countries. But, if information is easily available, it is nevertheless possible to record it in the comment fields of the geographic part (see Section 4.3.5). Note that after discussion, the consideration of the subdivisions of the current Yugoslavia has been rejected, although Euro+Med PlantBase consider them.

3.3.2 Subdivisions of Russia[13]

Russia is a large country and is not completely in Europe as defined above. It has been decided to divide it. Initial reports have established three main division from North to South [below in brackets and small letters], but the TDWG standards has established five ones which may be combined afterwards to compose these initial subdivisions (with few exceptions marked with a star *)

For a better comprehension of the limits inside Russia, we include two maps in Appendix 4.

3.3.2.1 Three small territories

- Kaliningradskaya Oblast' (between Poland and Lithuania)
- Novaya Zemlya
- Franz Josef Land (excl. Ushakova I. and Vize I.)

3.3.2.2 Five main subdivisions

At least we would prefer species being distributed through all five subdivisions. The lists of the territories included in these subdivisions are given below just to have a precise idea of the content of those subdivisions and are not to be used (but see the Encoding section below for non-recognised subdivisions in FaEu).

---

[13] NB: the romanization of administrative territory names follows the BGN/PCGN 1947 system, as given in ISO 3166-2 standard. Franz Josef Land being an island is still in English.

3.3.2.2.1 North European Russia (from west to east) [= Northern European Russia]
Southern boundary: approximately along 60°N
Are included:
- Murmanskaya Oblast'
- Kareliya Respublika
- Vologodskaya Oblast' *
- Arkhangel'skaya Oblast' mainland (incl. Nenetskiy Avtonomnyy Okrug, excl. Nova Zemlya and Franz Josef Land)
- Komi Respublika

[Central European Russia; Southern boundary: approximately along 52°N]

3.3.2.2.2 Northwest European Russia (from north to south)
Are included:
- Leningradskaya Oblast'
- Novgorodskaya Oblast'
- Pskovskaya Oblast'

3.3.2.2.3 Central European Russia (from west to east, north to south)
Are included:
- Tverskaya Oblast'[14]
- Yarovslavskaya Oblast'
- Kostromskaya Oblast'
- Smolenskaya Oblast'
- Moskovskaya Oblast'
- Vladimirskaya Oblast'
- Ivanovskaya Oblast'
- Nizhegorodskaya Oblast'
- Kaluzhskaya Oblast'
- Tul'skaya Oblast'
- Ryazanskaya Oblast'
- Mordoviya Respublika
- Chuvashkaya Respublika
- Bryanskaya Oblast'
- Orlovskaya Oblast'
- Lipetskaya Oblast'
- Tambovskaya Oblast'
- Penzenskaya Oblast'
- Ul'yanovskaya Oblast'
- Kurskaya Oblast' *
- Belgorodskaya Oblast' *
- Voronezhskaya Oblast' *

[14] New official name of ex-USSR Kalininskaya Oblast'; Tver replaces Kalinin for the city.

3.3.2.2.4 East European Russia (from west to east, north to south)

Are included:

- Kirovskaya Oblast'
- Udmurtskaya Respublika
- Permskaya Oblast' (incl. Komi-Permyatskiy Avtonomnyy Okrug)
- Mariy El Respublika
- Tatarstan Respublika
- Bashkortostan Respublika
- Samarskaya Oblast' *
- Orenburgskaya Oblast' *

3.3.2.2.5 South European Russia (from north to south) [=South European Russia]

Southern boundary: approximately along 46°N

Caucasian territories are excluded: Krasnodarskiy Kray, Stavropol'skiy Kray, Adygeya Respublika, Karachayevo-Cherkesskaya Respublika, Kabardino-Balkarskaya Respublika, Severnaya Osetiya Respublika, Chechenskaya Respublika, Ingushskaya Respublika, Dagestan Respublika

Are included:

- Saratovskaya Oblast'
- Volgogradskaya Oblast'
- Rostovskaya Oblast'
- Kalmykiya Respublika
- Astrakhanskaya Oblast'

4 Encoding entry and import format

4.1 Principles

The coding of the geopolitical units follows the principles hereafter in a sequential priority order:

(a) ISO 3166-1 gives a two letter code for the country level.

(b) TDWG gives a three or two letter code for the subdivision level.

(c) TDWG provides a system to gather units at a supra-country level, if necessary.

(d) ISO 3166-3 gives a four letter code for the former countries (like former Yugoslavia and Czechoslovakia), if necessary, some have been created for FaEu puposes (see table 2).

(e) Special cases, subdivisions without code: both Faroe Is. and Gibraltar have an ISO 3166-1 code, and not an ISO 3166-2 subdivision code respectively in Denmark and Great-Britain, although they are (still) dependent from these countries. As they are far away from their mainland, their code is only the ISO 3166-1 one.

(f) Special case, subdivision with an adding country code: Svalbard & Jan Mayen have each an ISO 3166-2 subdivision code in Norway, and an ISO 3166-1 country code. The adapted TDWG code is used.

In all cases, if the spelling is different between ISO and TDWG, ISO has priority.

Note that through the TDWG standard, the FaEu geographic codes are compatible with the Euro+Med PlantBase ones even if the latter provides more subdivisions in some areas like Yugoslavia.

4.2 General principles for the FaEu geopolitical level

What is in the contract is the country level species distribution, some of these countries being replaced by a partition of few subdivisions, as stated above. Still, the country level seems to be relevant for the end-users. Already we have two levels, and moreover, because standards exist, we allow to enter more precise distribution, but we have to be very clear about the meaning of the entries.

Let's take the example of France (FR), French mainland (FR-FRA), Corsica (FR-COR).

rec#	FR-FRA	FR-COR
1	P	A
2	P	
3	A	P
4		P
5	P	P
6	A	A

These records mean that a species has been recorded in French mainland (rec#1 & 2), in Corsica (rec#3 & 4), in both (meaning in whole France; rec#5), or has not been recorded from either (i.e., not recorded from political France; rec#6). Note that rec#1 & 3 are not equivalent to rec#2 & 4 respectively: for rec#1 & 3, the record/no record of the species has been checked for both subdivisions, and consequently marked as “A”, whereas for rec#2 & 4, it has not been checked. Therefore, they have not the same meaning.

See also main text, Section 3.1

4.3 Difficulties

PLEASE, READ CAREFULLY THE FOLLOWING

Note that the types of records required for the contract are rec#1, 3, 5 and 6 given above. It is possible to limit oneself to these types of records.

The following possibilities are ONLY **provided in order to facilitate the work process, to allow the expression of doubt and uncertainty, to allow temporary decisions until an eventual verification possibly long or difficult, to enter data because they are easily available, etc.**

If one does not feel easy to use this, please contact us, or skip these possibilities.

4.3.1 Subspecies

As a general principle, the subspecies distributions are entered separately from the species distributions that must be entered as well.

Two problems may arise:

- one of the subspecies may not be recorded in FaEu area; it must not be inserted, but an extra-limital comment should be entered for the species corresponding to the concerned area, possibly with the indication of the subspecies concerned in the comment field *Extra-Codes*.
- a species has been recorded in one of the geopolitical unit, but without precision of the subspecies (for example before that the species had been split into subspecies). Then the record should be entered under the species (and not under the nominal subspecies only inserted for the circumstance), unless it has been proved afterwards that this species record corresponds to one of the subspecies.

The distribution of the species as a whole within the FaEu area should be equivalent to the merge of the FaEu subspecies distributions, except in the second case above where the distribution will be the one entered under the species (see example below). The rule is that if a subspecies is fully or doubtfully recorded in a geopolitical unit, the species has consequently at least the same status and has the status of the subspecies that has the highest record status (i.e., P>P?>A). The validation process will check the consistency of these data.

Let's explain the following examples where *G1 sp1* a species with 2 subspecies recorded in the FaEu area, namely *G1 sp1 ssp1* and *G1 sp1 ssp2* (note that the nominal subspecies, *G1 sp1 ssp1* has been recorded in the area), where *G2 sp3* is a species with 2 subspecies recorded in the FaEu area, namely *G2 sp3 ssp4* and *G2 sp3 ssp5* (note here that the nominal subspecies, *G2 sp3 ssp3* has not been recorded in the FaEu area).

Note that columns and line in italics are errors.

rec#	Genus	species	Sub-Species	EE	FR-FRA	FR-COR	IT-ITA	IT-SAR	IT-SI	*SI*	*SL*	*SM*
7	G1	sp1		P	P	P	P?	P?	A	*A*	*A*	
8	G1	sp1	ssp1	P	P	P	P?	P?	A	*P*	*A*	*A*
9	G1	sp1	ssp2	P	P?	A	P?	A	A	*A*	*P?*	*P*
10	G2	sp3		[P]	[P?]	[A]		P	P			
11	G2	sp3	ssp4					P?	A			
12	G2	sp3	ssp5	P	P?	A		A	A			
13	*G2*	*sp3*	*ssp3*						*P*			

Rec#7 to 9 about *G1 sp1*.

The column EE means that *ssp1* and *spp2* have been recorded in Estonia, thus that *sp1* has been recorded in Estonia.

The column FR-FRA means that *ssp1* has been recorded in French mainland, and *ssp2* doubtfully, thus that *sp1* has been recorded in French mainland.

The column FR-COR means that *ssp1* has been recorded in French mainland, and not *ssp2*, thus that *sp1* has been recorded in Corsica.

The column IT-ITA means that *ssp1* and *ssp2* have been doubtfully recorded in Italian mainland, thus that *sp1* has been doubtfully recorded in Italian mainland.

The column IT-SAR means that *ssp1* has been doubtfully recorded in Sardinia, and not *ssp2*, thus that *sp1* has been doubtfully recorded in Sardinia.

The column IT-SI means that *ssp1* and *ssp2* have not been recorded in Sicily, thus that *sp1* has not been recorded in Sicily.

For the columns SI, SL, SM, an error will be notified in a report to GC during the validation process.

Rec#10 to 13 about *G2 sp3*. Note that the italicised brackets are not to be entered: they are just here for explanation, indicating an empty cell during the data entry process, or the status between brackets at the end of the data process.

The column EE means that *ssp4* has not been treated for Estonia (during the data entry process) or there is no information available (at the end of the data entry process), and *spp5* has been recorded in Estonia, thus that *sp3* has not been treated entirely for Estonia (during the data entry process), or has been recorded in Estonia (at the end of the data entry process).

The column FR-FRA means that *ssp4* has not been treated for French mainland (during the data entry process) or there is no information available (at the end of the data entry process), and *spp5* has been doubtfully recorded in French mainland, thus that *sp3* has not been treated entirely for French mainland (during the data entry process), or has been doubtfully recorded in French mainland (at the end of the data entry process).

The column FR-COR means that *ssp4* has not been treated for Corsica (during the data entry process) or there is no information available (at the end of the data entry process), and *spp5* has not been recorded in Corsica, thus that *sp3* has not been treated entirely for Corsica (during the data entry process), or has not been recorded in Corsica (at the end of the data entry process).

The column IT-ITA means that *ssp4* and *ssp5* have not been treated for Italian mainland (during the data entry process) or there is no information available (at the end of the data entry process), thus that *sp3* has not been treated for Italian mainland (during the data entry process), or there is no information available for Italian mainland (at the end of the data entry process).

The column IT-SAR means that *ssp4* has been doubtfully recorded in Sardinia, *spp5* has not been recorded in Sardinia, but that *sp3* has been recorded in Sardinia as a species but not as one of the two subspecies *ssp4* and *ssp5*, nor any other subspecies.

The column IT-SI means that *ssp4* and *ssp5* have not been recorded in Sicily, but that *sp3* has been recorded in Sicily as a species but not as one of the two subspecies *ssp4* and *ssp5*, nor any other subspecies. In that case, note that it would be an error to create a record such as rec#13 with the nominal subspecies *ssp3* that has not been recorded in FaEu area.

For the extra-limital codes, let's have *G3 sp6* with only one subspecies recorded in the FaEu Area, namely *G3 sp6 ssp7*.

rec#	Genus	species	Sub-species	FR-FRA	NAF	Extra-Codes
14	G2	sp6		P	P	NAF: G2 sp6 ssp6
15	G2	sp6	ssp7	P		

These two records means that the *ssp7* has been recorded in French mainland, thus the species *G2 sp6* as well, and that it has been recorded in North Africa as the subspecies *ssp6*, the nominal subspecies for instance.

Examples are also given in the table Appendix 2.

4.3.2 Country and subdivision level (= FaEu geopolitical units)

If references to literature are included in the comment fields, please follow the instructions in Chapter II Section References.

4.3.2.1 Records in a subdivided country

In the following, France (FR), French mainland (FR-FRA) and Corsica (FR-COR) are taken as examples.

A species must not be considered as recorded in subdivisions only because they have been recorded at a national country level. Nevertheless, we propose a way to record this condition, if checking is to be done later on for instance.

rec#	FR-FRA	FR-COR	Extra-codes
16			FR

This record means that the species has been recorded at least in one locality in France. The empty cells under FR-FRA and FR-COR mean that the distribution at the subdivision level has not been checked and that the species may have been recorded in French mainland or in Corsica or both.

Note that if several codes are entered in the Extra-codes field, they must be separated by a comma and a space.

Example: FR, IT, GR

rec#	FR-FRA	FR-COR	Extra-codes
16	P	A	FR
17	P		FR
18	A	P	FR
19		P	FR
20	P	P	FR

In these records, FR should not appear in the *Extra-codes* field. An error will be notified in a report to GC during the validation process. The code FR should be removed. The records will be then the same as rec#1 to 5 respectively.

4.3.2.2 Doubtful records

Encoding procedures for uncertainty are explained here, but it is stressed here that some complex possibilities can be skipped if they are felt as not necessary or to complicated by the Taxonomic Specialists (TSs) and Group Coordinators (GCs).

rec#	FR-FRA	FR-COR
21	P?	A
22	P?	
23	A	P?
24		P?
25	P?	P?

These records have the same broad meaning as rec#1 to 5 except that the record is doubtful for any reason. This reason should be commented (and referenced if possible; see section II for reference citation format in the *Fine distribution* field).

Note that P?=A? but that P? must be used. A? would be replaced automatically by P? during the validation process.

Note that the doubt or uncertainty (question mark) is not equivalent to the lack of checking (empty cell). In the first case, the specialist has checked the record/no record, but the definitive conclusion cannot be taken for any reason (possible misidentification; uncertainty of the location; reference to a work known not to be at a correct scientific level; species not seen since the year 1600 but not specifically searched in the area; date to be entered if known; etc.).

rec#	FR-FRA	FR-COR	Extra-codes
26			FR?

This type of record should be kept at a low percentage of the total.

rec#	FR-FRA	FR-COR	Extra-codes
27	P		FR?
28	P	A	FR?
29	P	P?	FR?
30	P?		FR?
31	P?	A	FR?
32		P	FR?
33	A	P	FR?
34	P?	P	FR?
35		P?	FR?
36	A	P?	FR?
37	P	P	FR?
38	P?	P?	FR?

In these records, FR? should not appear in the *Extra-codes* field. An error will be notified in a report to GC during the validation process. The code FR should be removed. The records will be then the same as records previously mentioned.

Note that rec#30 & 35 are not equivalent to rec#31 & 36 respectively.

rec#	FR-FRA	FR-COR	Extra-codes
39	A	A	FR
40	A	A	FR?

In the these records, an error will be notified in a report to GC during the validation process. The codes FR and FR? should be removed.

4.3.2.3 Former countries

If for any reason, former countries and names have to be used, the ISO 3166-3 standard must be used for those that have codes (see Table 2).

Example: former Czechoslovakia (CS, now CSHH) is now subdivided in Czech Republic (CZ) and Slovakia (SK).

rec#	CZ	SK	Extra-codes
41			CSHH

This record has the same meaning as rec#13. Precisely in this case, the species has been previously recorded in a check-list for the former Czechoslovakia, and verification is needed to precise in which new country (maybe both).

4.3.3 Extra-limital areas

During the meeting of the Advisory Team on Geographic Information in Padua (Alonso-Zarazaga and Bailly, 2000), it has been advocated that there should be a possibility to enter broad distribution data outside Europe, especially to determine the (non)-endemic status, see Section 4.3.4 below. As we do not require contractually that the extra-limital information is entered, it should be considered as a non-mandatory facility that can be useful in certain cases.

As it is a facility, note that certain rules applied to FaEu area are not to be followed if it is not necessary, but it is possible. For example, it is possible but not necessary to mark “A” for not recorded taxon at the end of data entry process in FaEu extra-codes.

It is possible to enter supra-country levels in two ways:

- using a system of 8 broad biogeographical distribution defined during the Padua meeting (see table 3). This system gives no precise boundaries, thus does not strictly follow administrative limits. It means that outside Europe, countries may belong to one or several units. They should be entered in separate columns (Extra-limital distribution part of the table in Appendix 2) as for the other FaEu codes, but mainly with status "P"; it is worthwhile not to use "P?" and "A", an empty cell there meaning nothing but not useful;
- using the Extra-Codes comment fields with other code systems at continental and sub-continental levels, e.g., codes of former countries like former Yugoslavia and Czechoslovakia (see Table 2), ISO codes, FAO codes for continents, TDWG codes for regions (see Table 4), …; note that the latter system follows the administrative limits, thus gathers strictly the units in a hierarchy (biogeopolitical system).

In both cases, although it is clear that they can be useful for requests, it is not clear how they can be used to enter data. In the examples above, putting FR in the *Extra-codes* field allows to have the minimum information which is required at the country level for FaEu contract. But putting the TDWG supra-country code SPA (which groups Spanish mainland, Andorra and Gibraltar) or 12 for South-western Europe adds no information for FaEu purpose but uncertainty, as we do not know in which country the species actually occurs. We could change the rules of signification for these supra-country codes (for example the species occurs in all included units), but it seems too complex to be tried.

Nevertheless, it may happen that it helps the work process, and then this possibility is provided although it should be used with precaution.

4.3.4 Continental level and endemicity

We have provided above two non-exclusive systems to indicate extra-limital distribution, depending on how the information is easily available. The endemic status could be calculated from the information available, whatever the system is used. Actually, because we do not require strictly that the extra-limital information is entered, we can only calculate with certainty the non-endemic status: when at least one extra-limital code is entered, the taxon is not endemic to FaEu area.

For those species where no extra-limital information is entered at all, it could be only a guess (for example if one native species has only been recorded in Belgium, it may be considered as endemic to FaEu area). The calculation will be let under the responsibility of the users for the current project. It may be part of a future proposal.

4.3.5 Infra-country level other than subdivisions recognised

If the data are easily available, or of particular interest, the *Extra-codes* field can be used to enter subdivision codes which are not recognised by FaEu coding system, especially codes from ISO 3166-2 or TDWG standards. If other systems are to be used, it must be indicated as well in this comment field. At least, one extra-FaEu location should be registered to state the non-endemic status.

In the following, Lavezzi Is. (no code) are small islands south of Corsica.

rec#	FR-FRA	FR-COR	Extra-codes	Fine distribution
42	A	P		FR-COR only in Lavezzi Is.
43		P		FR-COR only in Lavezzi Is.
44	A	P?		FR-COR maybe in Lavezzi Is.
45		P?		FR-COR maybe in Lavezzi Is.
46	A	P		FR-COR maybe in Lavezzi Is.
47		P		FR-COR maybe in Lavezzi Is.
48	A	P		FR-COR not in Lavezzi Is.
49		P		FR-COR not in Lavezzi Is.

The rec#42 & 43 mean that the species has been recorded only in Lavezzi Is. in Corsica, doubtfully for the rec#44 & 45 for which a comment should be added to explain the question mark. Note that the rec#44 & 45 have not the same meaning as the rec#46 & 47: the former mean that the species if recorded in Corsica is doubtfully recorded only in Lavezzi Is; the latter mean that the species is recorded in Corsica, but doubtfully not recorded from Lavezzi Is.

The rec#48 mean that the species has been recorded (doubtfully for rec#49) in Corsica but not in Lavezzi Is.

In the following, a species has been recorded in two republics in Russia, Chuvashkaya Respublika, code RU-CU (in Russia Central, code RU-RUC) and in Mariy El Respublika, code RU-ME (in Russia East, code RU-RUE).

rec#	RU-RUC	RU-RUE	Extra-codes
50	P	P	RU-CU, RU-ME

Note that the record means that the species has been recorded in these republics, but it does not mean that it has never been recorded in other territories belonging to RU-RUC and RU-RUE. The encoding should be then either of the following:

rec#	RU-RUC	RU-RUE	Extra-codes	Fine distribution
51	P	P	RU-CU, RU-ME	RU-RUC only in RU-CU<br>RU-RUE only in RU-ME
52	P	P		RU-RUC only in RU-CU<br>RU-RUE only in RU-ME

In the rec#52, an error will be notified in a report to GC during the validation process. It should be transformed in rec#51.

Again, it is a possibility, which is not required, but may be of interest in some cases.

4.4 From lower to higher levels, not the contrary

It is obvious that lower levels of distribution allows the reconstruction of distribution at the higher levels. Conversely, it is not possible to deduce automatically a species-list of a subdivision from a country level for instance, which means that answers to searches will be relevant only for the requested level. For example, if a species has been recorded in France, but with no precision if it has been recorded in mainland and in Corsica, it is not possible to deduce automatically that the species has been recorded in these two subdivisions; checking is needed there.

Thus, the species must not be considered as recorded in subdivisions only because they have been recorded at a national country level. We have proposed above a solution to record this condition (see section 4.3.2.1), if checking is to be done later on for instance. So, if a species list is needed for a given subdivision, it must be checked first if records have been looking for at this subdivision level for all the species.

The same condition may be also tricky for small countries (see above). Intelligent pre-programmed requests would probably help the end-users in both cases.

For any further discussion or information on geography in FaEu, please do not hesitate to contact the FaEu Paris Office (http://www.mnhn.fr/iga/membres/NB/FaEu, faunaeur@mnhn.fr), as well as the main FaEu site where documents are available for download (http://www.faunaeur.org/partners/partnerweb/alldocuments/alldocs1.html).

Additional references

Hollis S. & Brummitt R.K. (1992). World geographical scheme for recording plant distributions. Plant Taxonomic Database Standards n°2, version 1. International Working Group on Taxonomic Databases for Plant Sciences (TDWG). Pittsburgh: Carnegie Mellon University. ix+105 p.

Tutin, T.G. *et al.* (1964) Flora Europaea. Vol. 1: Lycopodiaceae to Platanaceae. Cambridge: Cambridge University Press,. xxxii + 464 pp. + 5 maps.

Table 1: Codes and names of basic geopolitical units recognised in FaEu area.

FaEUCode	FaEuName	Remarks
AD	Andorra	
AL	Albania	
AT	Austria	
BA	Bosnia and Herzegovina	
BE	Belgium	
BG	Bulgaria	
BY	Belarus	
CH	Switzerland	
CY	Cyprus	
CZ	Czech Republic	
DE	Germany	
DK-DEN	Danish mainland	Incl. Bornholm I.
DK-FOR	Faroe Is.	
EE	Estonia	
ES-BAL	Balearic Is.	Incl. Mallorca I., Menorca I., and Pityuses Is. (=Ibiza I. + Formentera I.)
ES-CNY	Canary Is.	
ES-SPA	Spanish mainland	Incl. Alboran I.
FI	Finland	
FR-COR	Corsica	
FR-FRA	French mainland	
GB-CI	Channel Is.	Incl. Jersey, Guernsey, Alderney
GB-GI	Gibraltar	
GB-GRB	Britain I.	Incl. Shetlands, Orkneys, Hebrides and Man Is.
GB-NI	Northern Ireland	
GR-AEG	Vóreion Aiyáion (North Aegean Is.)	Incl. Andípsara, Áyios Evstrátios, Foúrnoi, Ikaría, Khíos, Lésvos, Límnos, Oinoúsa, Psará, Sámos, Skópelos Kaloyeroi and other smaller islands
GR-CYC	Kikládes (Cyclades Is.)	Incl. Amorgós, Anáfi, Ánidros, Ándros, Andíparos, Denoúsa, Folégandros, Íos, Iráklia, Káros, Kímolos, Kéa, Kýthnos, Mílos, Mýkonos, Náxos, Páros, Políaigos, Sérifos, Sífnos, Síkinos, Sýros, Thíra, Tínos, Yiarós and other smaller islands
GR-DOD	Dodekánisos (Dodecanese Is.)	Incl. Alimniá, Árkoi, Astipálaia, Avgonísi, Ankathonísi, Farmakonísi, Ioinianísia, Kálimnos, Kalolímnos, Kandelioúsa, Kárpathos, Kásos, Khálki, Khamilí, Kínaros, Kos, Léros, Levítha, Lipsói, Meyísti, Nísiros, Ofidoúsa, Pátmos,

		Ródhos, Saría, Sími, Sírina, Tílos, Tría Nisiá, Yialí and other smaller islands
GR-GRC	Greek mainland	Incl. Andikíthira I., Evvia I., Ionian Is., Samothráki I., Northern Sporades Is., Thásos I.
GR-KRI	Kriti (Crete)	Incl. small adjacent islands like Gávdhos. Note that Andikíthira I. although being closer to Kriti than to mainland, belongs to a mainland province
HR	Croatia	
HU	Hungary	
IE	Ireland	
IS	Iceland	
IT-ITA	Italian mainland	
IT-SAR	Sardinia	
IT-SI	Sicily	Incl. adjacent Italian islands (Lipari Is., Ustica I., Egadi Is., Pantelleria I., Pelagie Is.)
LI	Liechtenstein	
LT	Lithuania	
LU	Luxembourg	
LV	Latvia	
MC	Monaco	
MD	Moldova, Republic of	
MK	Macedonia, the former Yugoslav Republic of	
MT	Malta	
NL	The Netherlands	
NO-NOR	Norwegian mainland	
NO-SVA	Svalbard & Jan Mayen	Incl. Bear I.
PL	Poland	
PT-AZO	Azores Is.	
PT-MDR	Madeira Is.	
PT-POR	Portuguese mainland	
PT-SEL	Selvagens Is.	
RO	Romania	
RU-FJL	Franz Josef Land	Excl. Ushakova I. and Vize I.
RU-KGD	Kaliningrad Region	
RU-NOZ	Novaya Zemlya	
RU-RUC	Central European Russia	
RU-RUE	East European Russia	
RU-RUN	North European Russia	
RU-RUS	South European Russia	
RU-RUW	Northwest European Russia	
SE	Sweden	Incl. Gotland I.
SI	Slovenia	

SK	Slovakia	
SM	San Marino	
TR-TUE	European Turkey	Incl. Imroz I. - Gökçeada, but not those in the Sea of Marmara
UA	Ukraine	
VA	Vatican City	
YU	Yugoslavia	Incl. Serbia, Kosovo, Voivodina, Montenegro

Table 2: ISO 3166-3 codes and names for former countries in the FaEu area.

ISO Code	ISO Name	Current status or name
BYAA	Byelorussian SSR	= Belarus
CSHH	Czechoslovakia	Incl. Czech Republic and Slovakia
DDDE	German Democratic Republic	In Germany
SUHH	USSR	Divided in several countries
	Not in ISO 3166-3<br>A previous code does not exist when one of the new country covers the former country	
FAEu Code	ISO Name	Current status
DEDE	West Germany	In Germany
YUYU	Yugoslavia (former)	Incl. Bosnia and Herzegovina, Croatia, Macedonia, Montenegro, Serbia, Slovenia

Table 3: Codes and names for broad regions outside FaEu area (biogeographical level) in Alonso-Zarazaga and Bailly (2000).

FaEUCode	FaEuName	Remarks
AFR	Afro-tropical region	
AUS	Australian region	
EPA	East Palaearctic	East of the border line here defined
NAF	North Africa	Not including Sinai Peninsula
NEA	Nearctic region	
NEO	Neotropical region	
NRE	Near East	Asian Turkey, Caucasian Russian republics, Georgia, Armenia, Azerbaijan, Lebanon, Syria, Israel, Jordan, Sinai Peninsula (Egypt), Arabian peninsula, Iran, Iraq
ORR	Oriental region	

Table 4: Codes and names for continent and region in TDWG system (Hollis and Brummitt, 1992).

Continent Code	Region Code	Continent Name	Region Name
1		Europe	
1	10	Europe	Northern Europe
1	11	Europe	Middle Europe
1	12	Europe	Southwestern Europe
1	13	Europe	Southeastern Europe
1	14	Europe	East Europe
2		Africa	
2	20	Africa	Northern Africa
2	21	Africa	Macaronesia
2	22	Africa	West Tropical Africa
2	23	Africa	West-Central Tropical Africa
2	24	Africa	Northeast Tropical Africa
2	25	Africa	East Tropical Africa
2	26	Africa	South Tropical Africa
2	27	Africa	Southern Africa
2	28	Africa	Middle Atlantic Ocean
2	29	Africa	Western Indian Ocean
3		Asia-Temperate	
3	30	Asia-Temperate	Siberia
3	31	Asia-Temperate	Russian Far East
3	32	Asia-Temperate	Middle Asia
3	33	Asia-Temperate	Caucasus
3	34	Asia-Temperate	Western Asia
3	35	Asia-Temperate	Arabian Peninsula
3	36	Asia-Temperate	China
3	37	Asia-Temperate	Mongolia
3	38	Asia-Temperate	Eastern Asia
4		Asia-Tropical	
4	40	Asia-Tropical	Indian Subcontinent
4	41	Asia-Tropical	Indo-China
4	42	Asia-Tropical	Malesia
4	43	Asia-Tropical	Papuasia
5		Australasia	
5	50	Australasia	Australia
5	51	Australasia	New Zealand
6		Pacific	
6	60	Pacific	Southwestern Pacific
6	61	Pacific	South-Central Pacific
6	62	Pacific	Northwestern Pacific

6	63	Pacific	North-Central Pacific
7		Northern America	
7	70	Northern America	Subarctic America
7	71	Northern America	Western Canada
7	72	Northern America	Eastern Canada
7	73	Northern America	Northwestern U.S.A.
7	74	Northern America	North-Central U.S.A.
7	75	Northern America	Northeastern U.S.A.
7	76	Northern America	Southwestern U.S.A.
7	77	Northern America	South-Central U.S.A.
7	78	Northern America	Southeastern U.S.A.
7	79	Northern America	Mexico
8		Southern America	
8	80	Southern America	Central America
8	81	Southern America	Caribbean
8	82	Southern America	Northern South America
8	83	Southern America	Western South America
8	84	Southern America	Brazil
8	85	Southern America	Southern South America
9		Antarctic	
9	90	Antarctic	Subantarctic Islands
9	91	Antarctic	Antarctic Continent

Appendix 4

Introduction

For a better comprehension of the limits inside Russia, we include two maps:

- Map 1 depicts the whole area of Fauna Europaea and the codes of the geo-political entities recognised hereby.
- Map 2 depicts subdivisions of Russia indicating the oblasts and autonomous republics within. In the following table, we will give the numbers identifying each of these in this map, their codes for Fauna Europaea and their names.

Three small territories

- Kaliningradskaya Oblast' (between Poland and Lithuania). Code RU-KGD
- Novaya Zemlya. Code RU-NOZ
- Franz Josef Land (excl. Ushakova I. and Vize I.). Code RU-FJL

North European Russia

Code RU-RUN

- 1 Murmanskaya Oblast'
- 2 Kareliya Respublika
- 3 Arkhangel'skaya Oblast' mainland (incl. Nenetskiy Avtonomnyy Okrug, excl. Nova Zemlya and Franz Josef Land)
- 4 Komi Respublika
- 5 Vologodskaya Oblast'

Northwest European Russia

Code RU-RUW

- 6 Leningradskaya Oblast'
- 7 Pskovskaya Oblast'
- 8 Novgorodskaya Oblast'

Central European Russia

Code RU-RUC

- 9 Kostromskaya Oblast'
- 10 Tverskaya Oblast'
- 11 Yarovslavskaya Oblast'
- 12 Ivanovskaya Oblast'
- 13 Nizhegorodskaya Oblast'
- 13a Vladimirskaya Oblast'
- 14 Smolenskaya Oblast'
- 15 Moskovskaya Oblast'
- 16 Ryazanskaya Oblast'
- 17 Mordoviya Respublika
- 18 Chuvashkaya Respublika
- 19 Ul'yanovskaya Oblast'
- 20 Kaluzhskaya Oblast'
- 21 Tul'skaya Oblast'
- 22 Lipetskaya Oblast'
- 23 Tambovskaya Oblast'
- 24 Penzenskaya Oblast'
- 25 Bryanskaya Oblast'
- 26 Orlovskaya Oblast'
- 27 Kurskaya Oblast'
- 28 Voronezhskaya Oblast'
- 29 Belgorodskaya Oblast'

East European Russia

Code RU-RUE

- 30 Kirovskaya Oblast'
- 31 Permskaya Oblast' (incl. Komi-Permyatskiy Avtonomnyy Okrug)
- 32 Udmurtskaya Respublika
- 33 Bashkortostan Respublika
- 34 Mariy El Respublika
- 35 Tatarstan Respublika
- 36 Samarskaya Oblast'
- 37 Orenburgskaya Oblast'

South European Russia

Code RU-RUS

- 38 Saratovskaya Oblast'
- 39 Volgogradskaya Oblast'
- 40 Astrakhanskaya Oblast'
- 41 Rostovskaya Oblast'
- 42 Kalmykiya Respublika

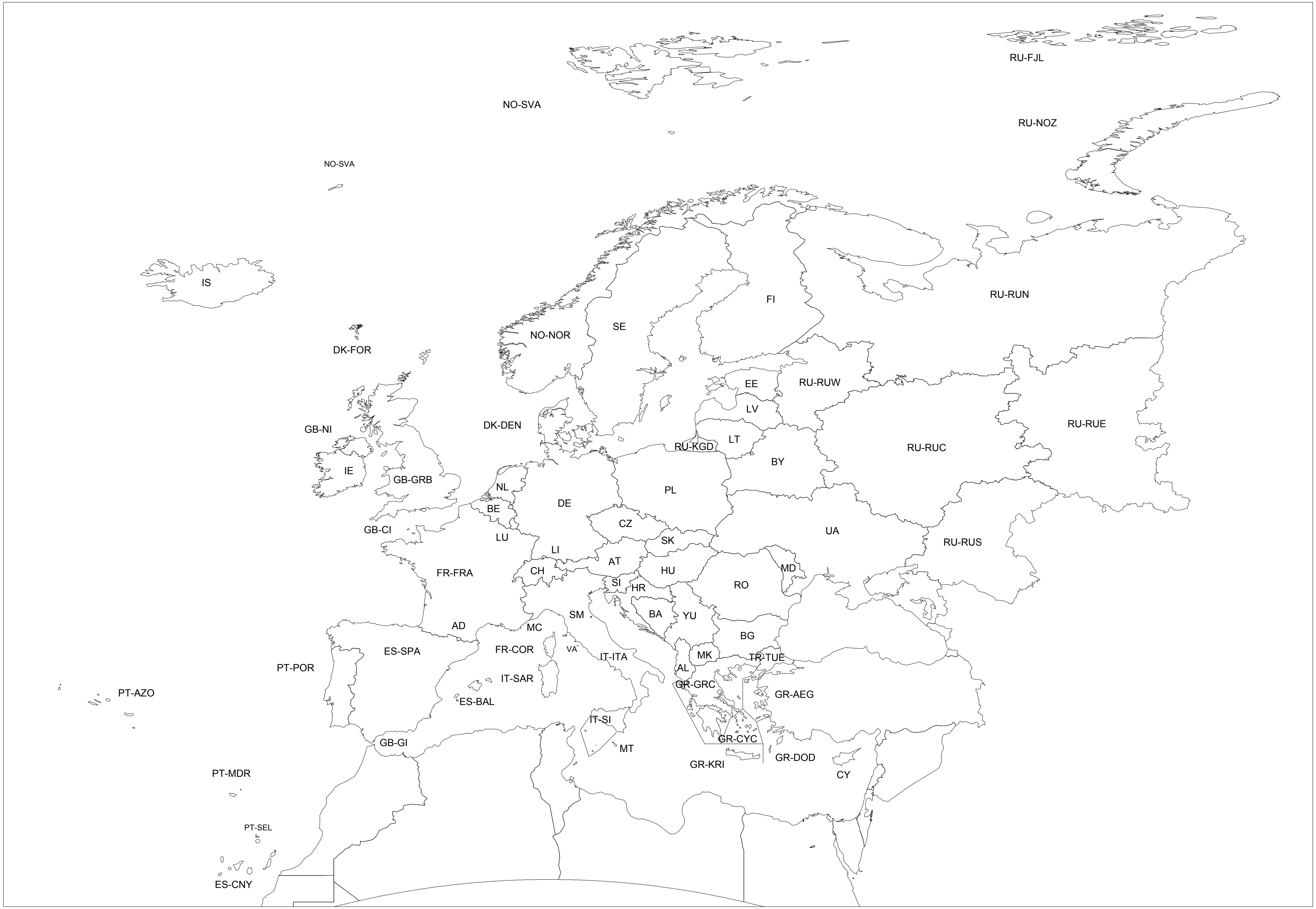

Map 1: Limits of Fauna Europaea and codes.

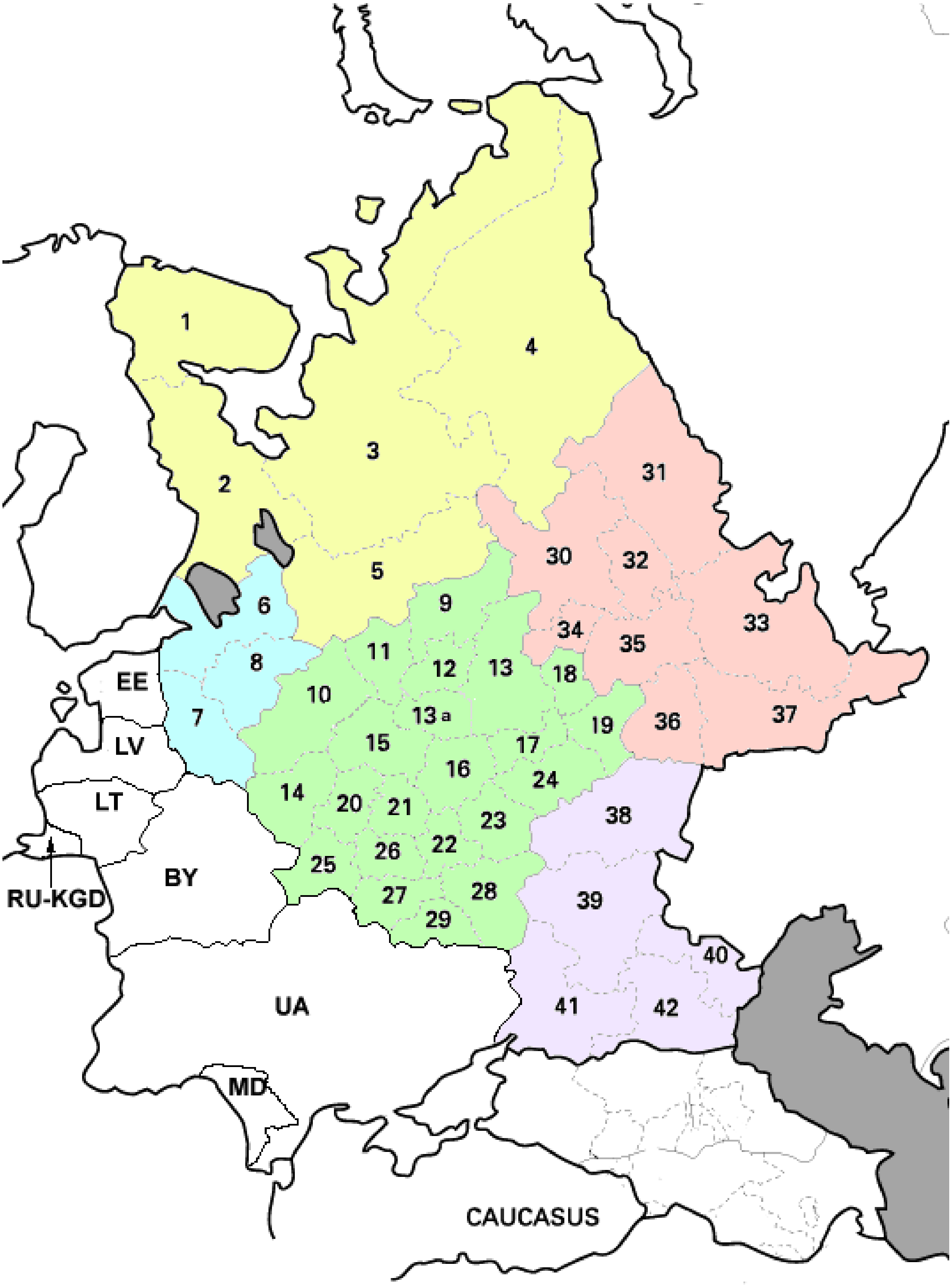

Map 2: Eastern Europe showing regions of Russia and their included oblasts and autonomous republics.
