# Supplementary figures and images for: Fauna Europaea – Orthopteroid orders
Source: Biodivers Data J. 2016 Jun 29;(4):e8905. doi: 10.3897/BDJ.4.e8905 (PMC5018117; doi:10.3897/BDJ.4.e8905)

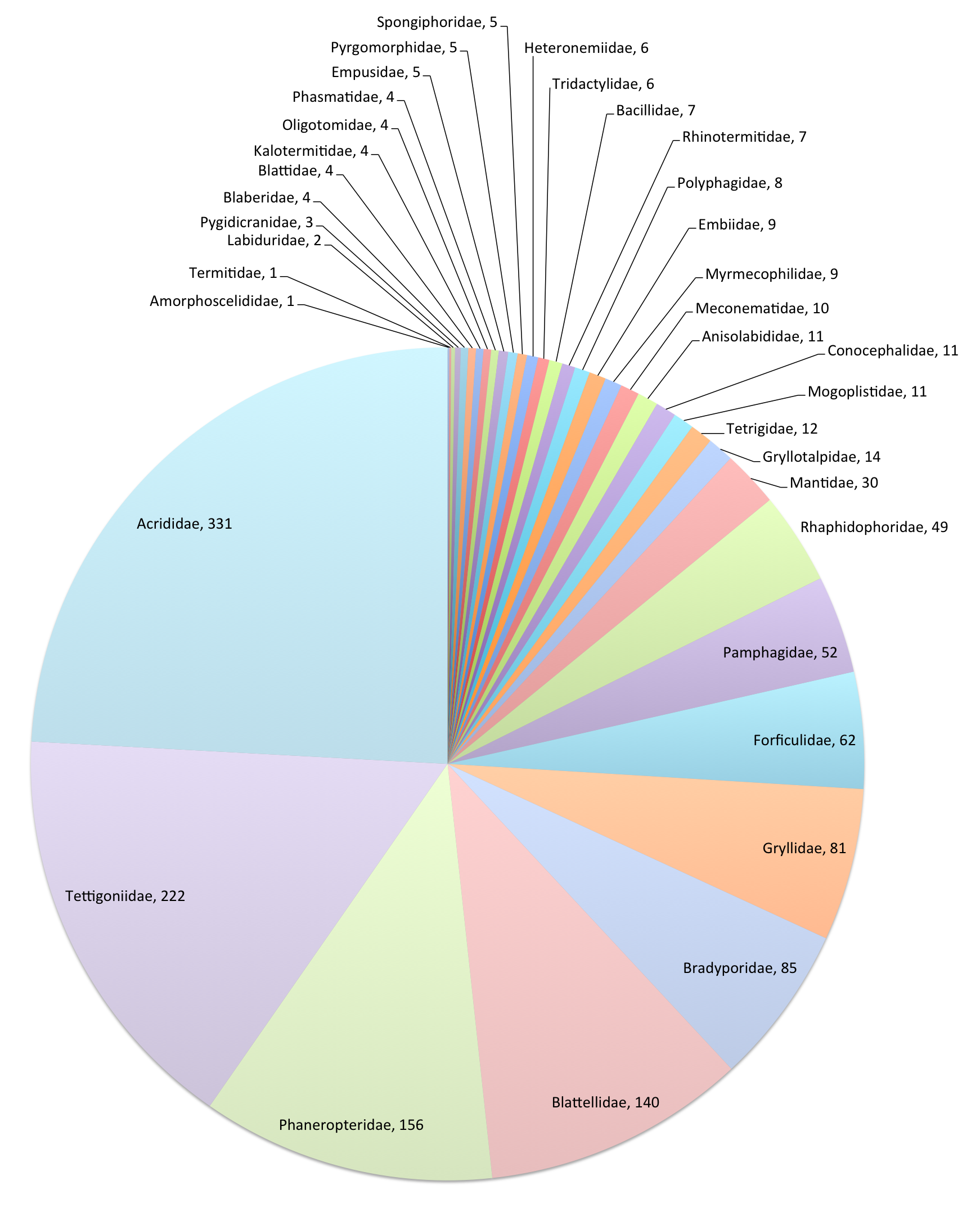

Supplement: Supplementary material 2 — FaEu Orthopteroid stats [file biodiversity_data_journal-4-e8905-s002.png]
